# Synthesis of β-Cyclodextrin-Functionalized Silver Nanoparticles and Their Application for Loading Cytisine and Its Phosphorus Derivative

**DOI:** 10.3390/molecules30061337

**Published:** 2025-03-17

**Authors:** Serik D. Fazylov, Oralgazy A. Nurkenov, Zhangeldy S. Nurmaganbetov, Akmaral Zh. Sarsenbekova, Ryszhan Ye. Bakirova, Olzhas T. Seilkhanov, Alexandr K. Sviderskiy, Ardak K. Syzdykov, Anel Zh. Mendibayeva

**Affiliations:** 1 Institute of Organic Synthesis and Coal Chemistry of the Republic of Kazakhstan, Karaganda 100008, Kazakhstan; iosu8990@mail.ru (S.D.F.); nzhangeldy@yandex.ru (Z.S.N.); ardak.syzdykov.96@inbox.ru (A.K.S.); anenyawa@mail.ru (A.Z.M.); 2 Department of Chemical Technology and Ecology, Karaganda Industrial University, Temirtau 101400, Kazakhstan; 3 Department of Physical and Analytical Chemistry, Karaganda University of the Name of E.A. Buketov, Karaganda 100074, Kazakhstan; chem_akmaral@mail.ru; 4 Department of Internal Diseases, Karaganda Medical University, Karaganda 100012, Kazakhstan; 5 Laboratory of Engineering Profile NMR-Spectroscopy, Sh. Ualikhanov Kokshetau University, Kokshetau 120000, Kazakhstan; seilkhanov@mail.ru; 6 Department of Mining, Metallurgy and Natural Sciences, Zhezkazgan University of the Name of O. Baikonurov, Zhezkazgan 100600, Kazakhstan; katsostud@mail.ru

**Keywords:** silver nanoparticles, cytisine, antiviral activity, β-cyclodextrin, inclusion complex, silver nanoparticles, encapsulation, nanocomposites, thermal decomposition

## Abstract

In this study, the synthesis and properties of β-cyclodextrin-functionalized silver nanoparticles and their loading with a drug component are considered. β-Cyclodextrin was used as a reducing agent and stabilizer in the preparation of silver nanoparticles. The use of β-CD-AgNPs in loading molecules of the alkaloid cytisine (Cz) and its O,O-dimethyl-N-cytisinilphosphate (CzP) derivative, which have pronounced antiviral properties, was studied. The formation of β-CD-Cz-AgNPs and β-CD-CzP-AgNPs was confirmed by UV spectroscopy and X-ray diffraction spectroscopy. Scanning electron microscopy and transmission electron microscopy showed that the obtained β-CD-Cz-AgNP and β-CD-CzP-AgNP nanocomposites were well dispersed with particle sizes in the range of 3–20 nm. ^1^H-, ^13^C-NMR and COSY, HMQC, HMBC and Fourier transform infrared spectroscopy revealed the reduction and encapsulation of AgNPs by β-Cz, and the TEM imaging results showed an increase in the size of nanoparticles after the introduction of cytisine and its phosphorus derivative. The kinetic parameters of the thermal degradation process of β-CD, Cz, CzP and their inclusion complexes Cz(CzP)-β-CD-AgNPs under isothermal conditions, which ensure the preservation of the kinetic triplet, were determined. The differences in the mechanism of thermal decomposition of the studied materials are described by the parameters of the Šesták–Berggren model (m and n), which demonstrated differences for different compounds: for β-CD, the values of the parameters m and n are 0.47 and 0.53, respectively, while for CzP-β-CD-AgNPs they reach values of 0.66 and 1.34. These results indicate differences in the mechanism of thermal decomposition of the studied materials.

## 1. Introduction

A promising synthon in the search for and creation of new antiviral drugs is the well-known alkaloid cytisine (Cz), contained in the seeds of the plant *Cytisus laborinum* L. and *Thermopsis lanceolata*, belonging to the legume family (*Fabaceae, Leguminosae*). Cytisine is a substance with a “ganglionic” effect and, due to its stimulating effect on breathing, is considered a respiratory analeptic [1,2]. The exact mechanism of the effect of Cz has not been examined to date. It is likely to have effects similar to varenicline, that is, to reduce the enjoyment of smoking during the prequit period and to reduce withdrawal discomfort after quitting [3].

Cz and its derivatives, according to numerous studies, can bind to adrenaline, dopamine, serotonin, histamine and muscarinic receptors. Cz can also be used in shock and collapse states, respiratory and circulatory depression in patients with infectious diseases, etc. [4]. Therefore, possible anti-inflammatory, antispasmodic, antiarrhythmic, antiviral and neurotropic properties of Cz are currently being widely studied [4,5,6,7]. Recently, new synthetic derivatives of Cz have shown cytotoxicity and virus-inhibitory activity against human respiratory viruses: influenza A viruses of the H1N1, H3N2, H5N2 subtypes, influenza *B viruses* of the *B/Yamagata* and *B/Victoria* lineages and human parainfluenza virus type 3; the effect of Cs and its derivatives on various stages of influenza virus reproduction has been studied [5,6]. The cytisinium-O,O-dimethyl phosphate (Czt) synthesized by us also showed a high antiviral effect against hepatitis B [7]. The new phosphorous derivative of cytisine showed low toxicity (LD_50_ 1800 mg/kg).

The above-mentioned physiological effects of Cz and its O,O-dimethylphosphate derivative (CzP) indicate the promise of studying them as effective inhibitors of COVID-19 virus proteases and the ACE2 receptor. However, the high toxicity of Cz (LD_50_, 2 mg/kg, intravenously in mice) is an obstacle to its widespread use in medical practice as a respiratory analeptic [6]. Gastrointestinal disorders (dyspepsia and nausea), headache, increased appetite, dry mouth and irritability have also been reported [8]. For this reason, there is a need to obtain encapsulated forms of Cz with improved biofunctional properties and compare them with the corresponding characteristics of CzP (prolonged release and low toxicity). Future studies should test these hypotheses.

The development of nanotechnology has made it possible to obtain nanomaterials with unique properties in biomedical fields due to their interesting biological properties [9,10,11]. Among them, there is great interest in silver nanoparticles (AgNPs) and materials using them mainly due to their unusual physical and bioactive properties [12,13,14]. Silver is a well-known antimicrobial agent; it is effective against bacteria, viruses, fungi and yeast, including a number of strains resistant to antibiotics. The biochemical properties of AgNPs are associated with a number of parameters, including size, shape and stability, which depend mainly on the methodology and conditions of their preparation [15,16]. These properties are significantly enhanced by using AgNP nanoparticles due to a sharp increase in surface area.

**Figure 1:**
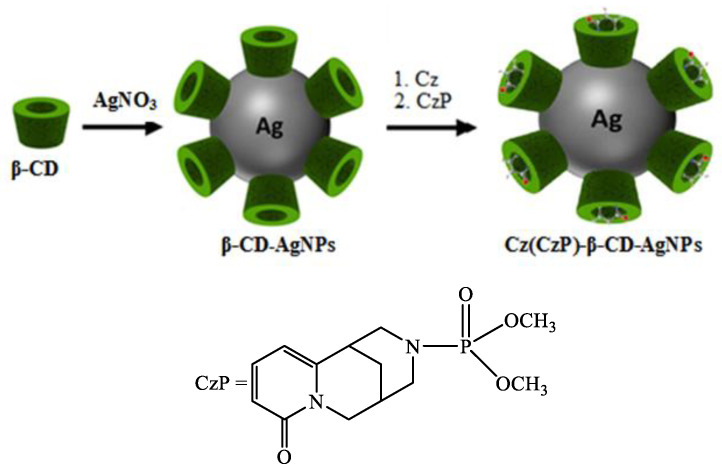
Schematic representation for the synthesis of Cz-β-CD-AgNPs and CzP-β-CD-AgNPs.

Cyclodextrins (α-, β- and γ-CD) are natural cyclic oligosaccharides formed by the degradation of natural starch. CD can serve as a good reagent for the reduction of metal salts and bind to the surface of nanoparticles by chemisorption. β-CD plays an important role in preventing the aggregation of nanoparticles, thus contributing to their stability in solution. For example, they have been successfully used in the preparation of Ag and Au nanoparticles [17,18,19,20,21] due to their good solubility in water [22,23,24,25]. A. Abu-Okeil et al. [26] used aminated β-CD to obtain AgNPs with particle sizes in the range of 1–9 nm. The hydrophobic cavity and unique ionic effects of cyclodextrins expand their application in drug delivery and solubilization [27,28,29,30]. In our previous study, we described the preparation and some features of the encapsulation mechanism of Cz with α-, β- and γ-CD [31,32]. The present study demonstrates the preparation and thermochemical characterization of encapsulated inclusion complexes of Cz and its phosphate derivative as organic ligands with AgNPs. The modified Cz(CzP)-AgNP compositions can be considered as a promising platform for improving the stability and expanding their potential in biomedical applications (Figure 1).

## 2. Results and Discussion

### 2.1. Description of Synthesis and Characterization of the Structure of Cz(Czt)/β-CD-AgNP Nanocomposites

The scientific literature describes many physico-chemical and “green green” methods for the production of AgNP nanocomposites involving various polymeric substances (PVP, PEG et al.) [33], oligosaccharides (α-, β- and γ-CD) [16,17,18,19,20,21], etc. CDs can be used in the production of nanocomposites and metal nanoparticles, because they can affect the characteristics of nanoparticles, such as drug loading, solubility, stability and bioavailability [33,34]. CDs have a unique ability to reduce Ag^+^ ions to metallic AgO and prevent agglomeration of the resulting AgNPs [34,35,36,37]. The method described here at the first stage included the reduction of the [Ag(NH_3_)_2_]^+^ complex to metallic AgO with an aqueous solution of β-CD. The initial concentrations of the reaction components were 0.003 mol/L and 0.05 mol/L for AgNO_3_ and the reducing agent, respectively. The ammonia concentration ranged from 0.005 to 0.01 mol/L. The color of the solution gradually changed from colorless to pale yellow, which intensified and subsequently changed to intensely yellow-brown, indicating the formation of AgNP nanoparticles [38,39]. With a further increase in the reaction time and pH of the solution, the color of the solution changed to dark brown, and then to gray. These observations show that as the reaction time increases, the particle size and aggregation of silver nanocrystals gradually increase. All measurements were carried out at room temperature (≈20 °C).

At the second stage, solutions of the Cz-β-CD-AgNP (**1**) and CzP-β-CD-AgNP (**2**) nanocomplexes were obtained by gradually adding Cz and CzP to the obtained β-CD-AgNP solution. Nanocomposites, which were dark brown powders soluble in water and DMSO, were isolated from the obtained colored solutions. Analyses of elemental data and atomic absorption spectroscopy showed that the silver content in nanocomposites (**1**) and (**2**) was 4.0 and 3.2%, respectively. All solutions were freshly prepared using double-distilled water and stored in the dark to avoid any photochemical reactions. All glassware used in the experimental procedures was cleaned with a fresh HNO_3_/HCl solution (3:1 by volume), thoroughly rinsed with double distilled water and dried before use.

### 2.2. Characterization of the Structure of Cz(Czt)/β-CD-AgNP Nanocomposites

**Figure 2:**
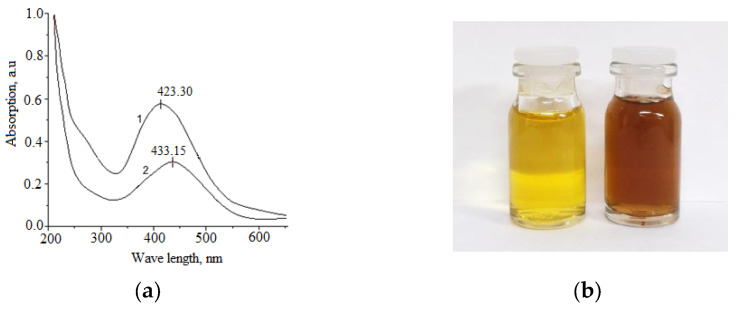
UV-vis spectrum of Cz-β-CD-AgNPs (**1**), CzP-β-CD-AgNPs (**2**) (**a**), the solution of optimum conditions (**b**).

In the UV spectra of Cz-β-CD-AgNP and CzP-β-CD-AgNP compounds, absorption peaks can characterize the effect of the surface plasmon resonance of nanoparticles, and the morphology of AgNPs correlates with the position of their maximum absorption peak. Figure 2 shows the absorption spectra of synthesized nanoparticles in the ultraviolet range.

**Figure 3:**
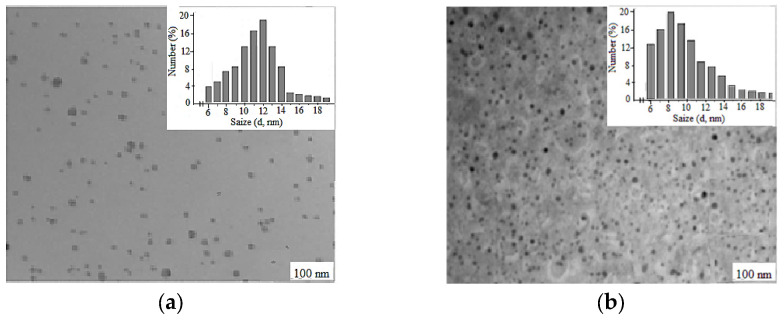
Electron micrographs of nanocomposites Cz-β-CD-AgNPs (**a**) and CzP-β-CD-AgNPs (**b**) and diagrams of silver nanoparticles’ size distribution in the β-cyclodextrin matrix.

The synthesized Cz(CzP)-β-CD-AgNPs showed a typical plasma band at wave lengths of 423.30 and 433.15 nm, which confirms the production of spherical nanoparticles of the composition [34,35]. TEM was performed to monitor the morphology, size and dispersion of the obtained Cz-β-CD-AgNPs. Figure 3 shows the TEM image which shows that Cz-β-CD-AgNP have a mostly subspherical shape. The histogram showed a narrow particle size distribution (in a solution with a pH of 9), since the particle size was concentrated in the range from 6 to 20 nm, with an average particle size of 11.78 ± 1.03 nm. In a solution with pH12, the nanoparticle size distribution is more narrowly dispersed, with a predominant number of nanoparticles (87%) in the range of 6–12 nm.

**Figure 4:**
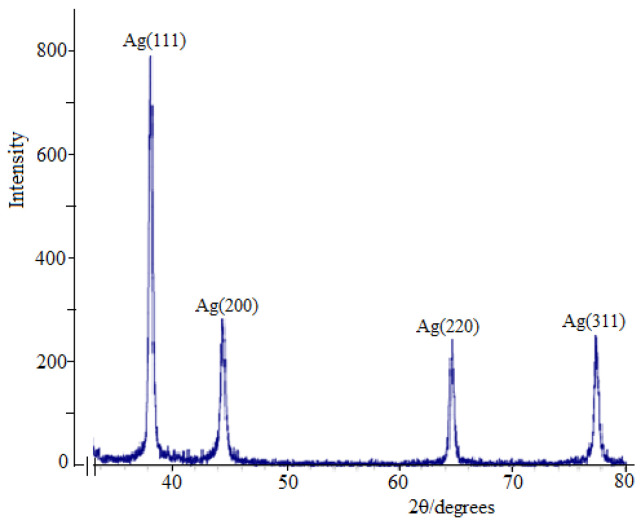
XRD pattern of β-CD-Cz-AgNPs.

The synthesized nanoparticles were also characterized by X-ray diffraction (XRD) (Figure 4). The peaks on the X-ray image confirmed that the AgNPs had a face-centered cubic shape, while the peaks of the contaminated crystalline phases could not be detected.

Figure 4 shows X-ray diffraction patterns of Ag-NPS, the resulting nanocomposite with Cz, which indicates the formation of the crystalline structure of silver. X-ray peaks in a wide range of angles of 2θ(30° < 2° < 80°) showed that peaks at 38.02°, 44.07°, 64.35° and 77.21° can be attributed to 111, 200, 220 and 311 crystalline structures of face-centered cubic (fcc) silver nanocrystals, respectively (Ag XRD Ref. № 00-004-0783) [35,36,37,38,39,40]. The intensity of the peaks and their clarity indicate that Cz-β-CD-AgNPs have a highly crystalline nature. It was also found that the intensities of 111, 200, 220 and 311 reflections due to the AgNPs phase increase along with an increase in AgNPs in the studied media. No other peaks were present as impurities were found on the X-ray images. Thus, these results provide clear evidence of the presence of AgNPs in the Cz-β-CD-AgNP composition.

**Figure 5:**
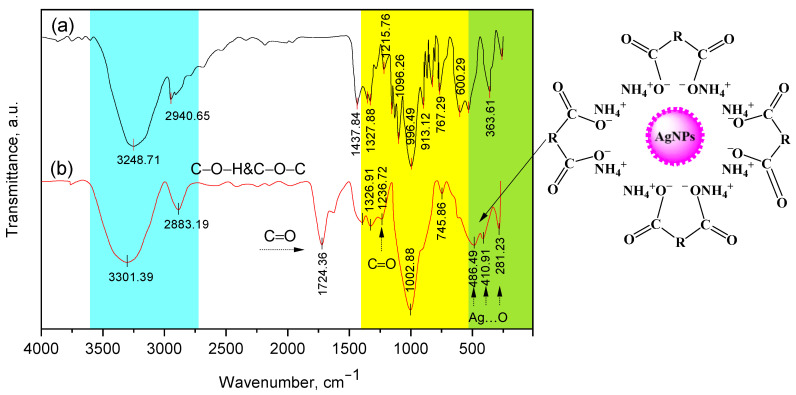
IR Fourier spectra for β-CD (a) and Cz−β-CD−AgNPs (b). The different colors in the figure show the characteristic areas of the absorption bands of various functional groups in the structure of compounds.

The interaction of the obtained β-CD-AgNPs with Cz and CzP was also confirmed by FT-IR spectra (Figure 5). The intense wide oscillation band observed at 3301 cm^−1^ is characterized by oscillations of the OH and NH groups, and at 2883 cm^−1^ there are oscillations of the C-H groups. In the region of 1724 cm^−1^, there is an intense new band characteristic of the C=O group of carboxylic acid. A wide oscillation band in the range from 3425 to 3223 cm^−1^ indicates the deprotonation of OH groups of β-CD in an alkaline solution, which facilitates the synthesis and stabilization of AgNPs [35,36,37,38]. Similar data are typical for Cz-β-CD-AgNPs. The bands caused by C-O fluctuations merged into a wide envelope band at 1328–1236 cm^−1^. A wide intense band at 1002 cm^−1^was caused by fluctuations in the C-O-C group of the glycoside bridge (Cz-AgNPs-β-CD). The aliphatic stretching of C-H at 1326–1236 cm^−1^ is caused by deformation vibrations of these groups (Figure 5) [37]. The deformation vibrational peak of the N–H CD group at 1570 cm^−1^ was slightly attenuated, suggesting that the amino group may interact with Ag^+^ and participate in the reduction and stabilization of AgNPs. The intensity of the peaks and their clarity indicate that Cz-β-CD-AgNPs have a highly crystalline nature. It was also found that the intensities of 111, 200, 220 and 311 reflections due to the AgNPs phase increase along with an increase in AgNPs in the studied media. No other peaks present as impurities were found on the X-ray images. Thus, these results provide clear evidence of the presence of AgNPs in the Cz-β-CD-AgNP composition.

The interaction of the obtained β-CD-AgNPs with Cz and CzP was also confirmed by FT-IR spectra (Figure 5). The intense wide oscillation band observed at 3301 cm^−1^ is characterized by oscillations of the OH and NH groups, and at 2883 cm^−1^ there are oscillations of the C-H groups. In the region of 1724 cm^−1^, there is an intense new band characteristic of the C=O group of carboxylic acid. A wide oscillation band in the range from 3425 to 3223 cm^−1^ indicates the deprotonation of OH groups of β-CD in an alkaline solution, which facilitates the synthesis and stabilization of AgNPs [35,36,37,38]. Similar data are typical for Cz-β-CD-AgNPs. The bands caused by C-O fluctuations merged into a wide envelope band at 1328–1236 cm^−1^. A wide intense band at 1002 cm^−1^was caused by fluctuations in the C-O-C group of the glycoside bridge (Cz-AgNPs β-CD). The aliphatic stretching of CH at 1326–1236 cm^−1^ was caused by deformation vibrations of these groups (Figure 5) [41]. The deformation vibrational peak of the NH group CD at 1570 cm^−1^ was slightly attenuated, suggesting that the amino group may interact with Ag^+^ and participate in the reduction and stabilization of AgNPs.

The spectral characteristics of the β-CD and cytisine inclusion complexes were previously characterized by ^1^H, ^13^C NMR spectroscopy and two-dimensional spectra of COSY (^1^H-^1^H) and HMQC (^1^H-^13^C) [30]. Analysis of these spectra showed that the greatest difference in the values of chemical shifts of protons in the cytisine-β-cyclodextrin inclusion complex is observed in H-3 (0.15 ppm) and H-5 (0.15 ppm) atoms. The formation of a nanocomposition of β-CD-AgNPs, Cz(CzP)β-CD and Cz(CzP)-β-CD-AgNPs was also studied using ^1^H, ^13^C NMR and COSY, HMQC, and HMBC spectroscopy (Figures S1a–c–6a–c). It should be noted that the nature of the spectrum of the complex is influenced by the shielding effect of the interaction of AgNPs with β-CD.

### 2.3. Thermogravimetric Analysis of Cz, CzP, Cz(CzP)-β-CD and Cz(CzP)-β-CD-AgNPs

**Figure 6:**
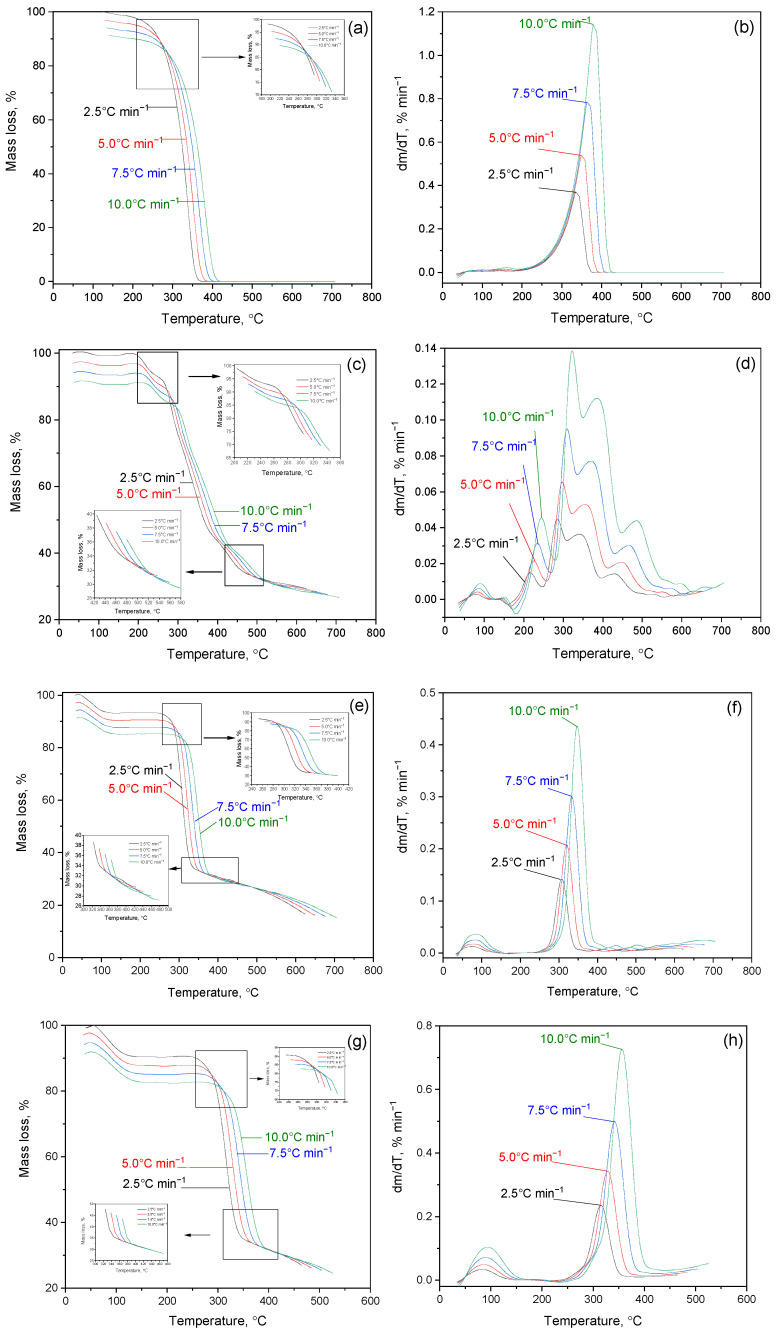

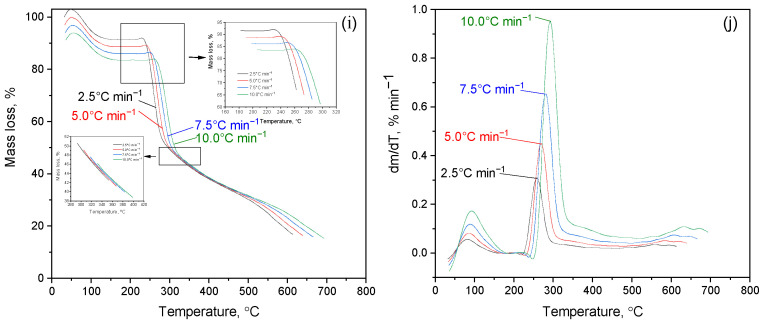
TG/DTG curves in nitrogen atmosphere: (**a**,**b**) Cz; (**c**,**d**) CzP; (**e**,**f**) Czβ-CD; (**g**,**h**) Cz-β-CD-AgNPs; (**i**,**j**) CzP-β-CD-AgNPs.

Figure 6 shows the results of a study of the kinetics of thermal decomposition of cytisine, cytaphate and their Cz-β-CD, Cz(CzP)-β-CD inclusion complexes and their nanocompositions modified with AgNP nanosilver. Figure 6a–d show thermograms of the thermal degradation of cytisine and cytaphate, respectively. The analysis of the TG curve on the presented thermograms indicates an intensive process of thermal decomposition of the studied objects. For Cz this process occurs at a temperature of T_term_(I) = 260–440 °C (Figure 6a), and for CzP at T_term_(I) = 240–500 °C (Figure 6c). A maximum is observed on the DTG curves (Figure 6b,d). The mass loss of Cz and CzP is almost completed at temperatures of 450–250 °C.

The initial period of thermal degradation of Cz-β-CD (1:1) at T_term_(I) = 100 °C is characterized by the removal of water located in the β-CD cavity (Figure 6e,f). As can be seen from Figure 6e,f, further heating of the Cz-β-CD inclusion complex leads to a mass loss (Δm = 65%), which begins at a temperature of T_term_(II) = 200–360 °C.

**Figure 7:**
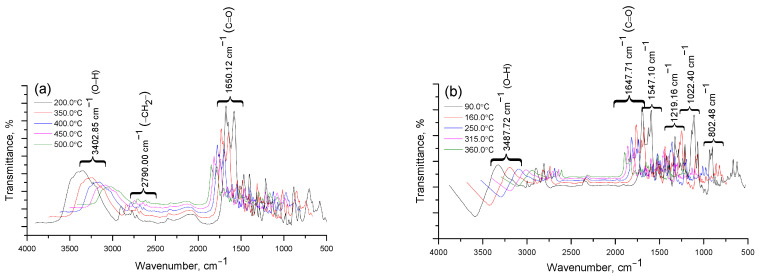

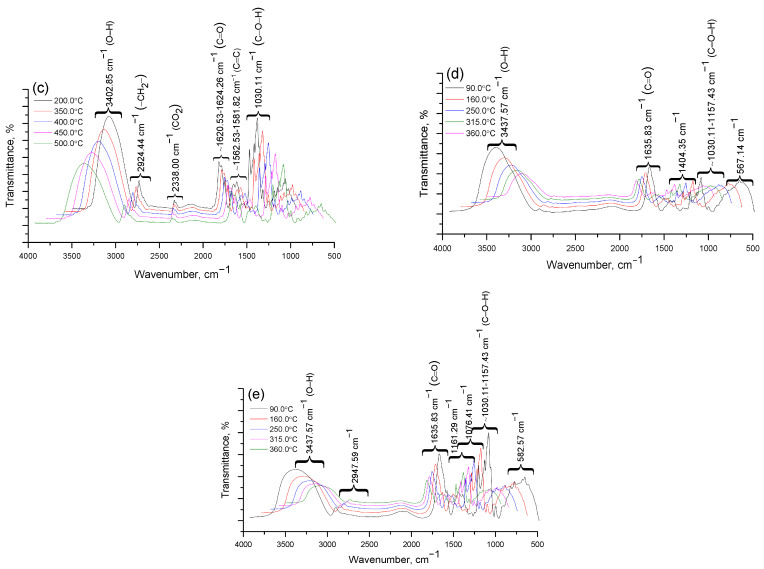
Results of IR spectroscopic analysis of degradation products Cz (**a**), CzP (**b**), Cz-β-CD (1:1) (**c**), Cz-β-CD-AgNPs (**d**), CzP-β-CD-AgNPs (**e**).

The result of this process is the formation of various fragments and destruction products caused by the destruction of the complex structure. Thermal destruction of the Cz-β-CD complex is subject to the mechanism of sequential rupture of bonds and the release of the components of the complex from the cavity of the β-CD molecule. For the inclusion complexes Cz(CzP)-β-CD modified with silver nanoparticles AgNPs, a similar behavior is observed at the initial stages of heating (Figure 6g–i). The first peak in the DTG curve (T_term_ (I) = 100 °C) is associated with the removal of water located inside the β-CD cavity. At T_term_ (II) = 280–440 °C, a sharp mass loss is observed in the TG curves (Δm = 74% for Cz-β-CD-AgNPs and Δm = 62% for CzP-β-CD-AgNPs), which corresponds to the final decomposition of the Cit(Citaf)-β-CD-AgNPs inclusion complexes. This subsequently leads to the destruction of the β-CD structure and the removal of volatile decomposition products. During further heating above 500 °C, stabilization of heat-resistant residues such as silver nanoparticles is observed (Figure 6i,j). These findings are also confirmed by the results of IR spectroscopic analysis (Figure 7a–e).

**Figure 8:**
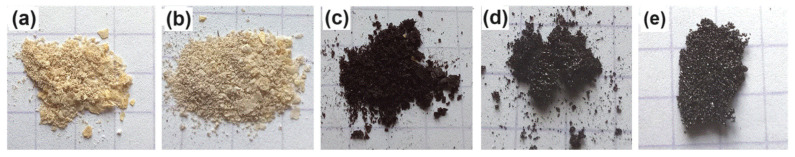
Annealed cytisine at different temperatures: 90 °C (**a**); 160 °C (**b**); 250 °C (**c**); 315 °C (**d**); 360 °C (**e**).

The following stages of the study present the results of annealing Cz and its derivatives. After heat treatment of Cz at a temperature of 90 °C, slight deformation of the structure is observed, which is accompanied by darkening of the material (Figure 8).

During the annealing of the material samples at a temperature of 160 °C, the formation of a relief is recorded, characterized by soft, smooth contours reminiscent of a steppe landscape. Further carbonization of cytisine leads to a significant change in color, expressed in a transition to a dark beige shade (Figure 8b). With an increase in temperature to 315 °C, an increased change in the microrelief of the surface of this substance is noted. This is expressed in the formation of local bulges and depressions, which imparts significant roughness to it (Figure 8c). These changes indicate the presence of intense transformations in the internal structure of the substance caused by thermal action. Annealing at a temperature of 350 °C leads to some smoothing of the surface relief; pronounced darkening and the appearance of metallic luster are observed (Figure 8d). With further annealing at 360 °C, obvious destruction and crumbling of the substance is recorded, which indicates almost complete disintegration of Cz (Figure 8c).

**Figure 9:**
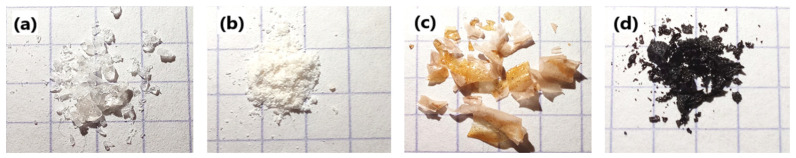
Annealed CzP at different temperatures: 90 °C (**a**), 160 °C (**b**), 250 °C (**c**), 360 °C (**d**).

The following photographs (Figure 9) show fragments of annealed CzP obtained at different heating temperatures. CzP annealed at a low temperature (90 °C) exhibits transparent crystals (Figure 9a). At a heating temperature of 160 °C, a more homogeneous white powder was obtained (Figure 9b). CzP annealed at a high temperature (250 °C) is distinguished by a yellowish color and a larger fraction. This indicates the initial stages of decomposition of the material (Figure 9c). The crystals appear to be quite large and unevenly colored, which may indicate the presence of different phases. CzP annealed at a high temperature of 360 °C is black and appears as a fine powder. This indicates significant transformations that occurred during the processing (Figure 9d).

**Figure 10:**
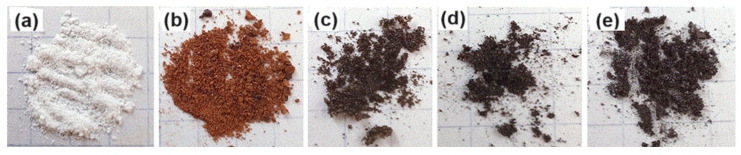
Annealed inclusion complex Cz-β-CD (1:1) at different temperatures: (**a**) 80 °C; (**b**) 270 °C; (**c**) 300 °C; (**d**) 350 °C; (**e**) 450 °C.

It is interesting to study how the characteristics of the inclusion complexes Cz-β-CD (1:1) and Cz-β-CD-AgNPs change during heat treatment and their further functionality in a nitrogen atmosphere. With increasing temperature, the mobility of the molecular chain, as well as the processes occurring during recrystallization of the inclusion complex, affect its morphology. After heat treatment at 80 °C, the relief of the inclusion complex Cz-β-CD is characterized by slight deformation with darkening (Figure 10a). The process of annealing the inclusion complex Cz-β-CD at a temperature of 270 °C is characterized by the formation of a wavy relief with local bumps and recesses, and subsequent charring leads to significant darkening of the material to a light brown shade (Figure 10b).

This phenomenon indicates the presence of intense transformations in the interior of the complex, as well as in its physicochemical properties, caused by thermal exposure. Such changes are associated with phase transformations or reorganization of the molecular structure of the Cz-β-CD inclusion complex [40]. With an increase in temperature to 300 °C, an enhanced change in the surface microrelief of the Cz-β-CD inclusion complex is observed, manifested by the formation of local bulges and depressions, which imparts roughness to the surface (Figure 10c). After annealing at 350 °C, the Cz-β-CD complex exhibits some smoothing of the surface relief, with pronounced darkening and metallic luster (Figure 10d). Annealing at 450 °C leads to the destruction and disintegration of the brittle clathrate, which indicates almost complete disintegration of the Cz-β-CD complex (Figure 10e).

**Figure 11:**
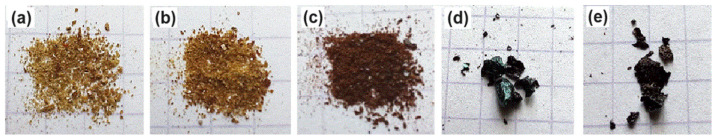
Annealed Cz-β-CD-AgNPs inclusion complex: (**a**) 90 °C; (**b**) 160 °C; (**c**) 250 °C; (**d**) 315 °C; (**e**) 360 °C.

Annealing of the Cz-β-CD-AgNPs nanocomposite was also carried out (Figure 11). After heat treatment at a temperature of 90 °C, slight deformation is observed on the surface of the Cz-β-CD-AgNPs composition, accompanied by darkening (Figure 11a). The process of annealing the complex at 160 °C leads to darkening of the material to a light brown shade, as shown in Figure 11b.

Annealing at 250 °C does not lead to any noticeable change in the surface morphology of the complex and is only characterized by darkening of the sample (Figure 11c). After annealing at 315 °C, some smoothing of the surface relief of the complex occurs, with darkening and metallic luster (Figure 11d). The membranes become brittle. Annealing at 360 °C leads to destruction and disintegration of the brittle complex, indicating almost complete decomposition of the Cz-β-CD-AgNPs system (Figure 11e).

**Figure 12:**
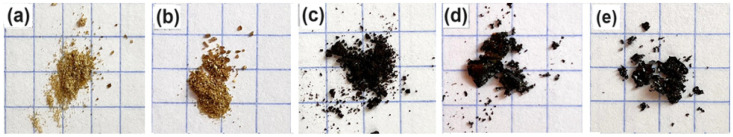
Annealed inclusion complex CzP-β-CD-AgNPs: (**a**) 90 °C; (**b**) 160 °C; (**c**) 250 °C; (**d**) 315 °C; (**e**) 360 °C.

Similar studies were also conducted for the inclusion complex CzP-β-CD-AgNPs. As the temperature increased from 90 °C to 160 °C, the crystals of the CzP-β-CD-AgNPs nanoclathrate began to change their color (Figure 12a).

When the temperature is increased to 160 °C, the CzP-β-CD-AgNPs clathrate complex loses bound water that may be present in the composite. This leads to shrinkage and compaction of the complex (Figure 12b). At temperatures of 250–360 °C, the organic components of the CzP-β-CD-AgNPs complex undergo decomposition, which causes darkening of the material. This is due to the processes of degradation and caramelization of the sugar residues of β-cyclodextrin (Figure 12c). Also, the change in the color of the material is due to the agglomeration of oxidized silver particles at high temperatures, which leads to the appearance of a dark or black shade (Figure 12c–e).

**Figure 13:**
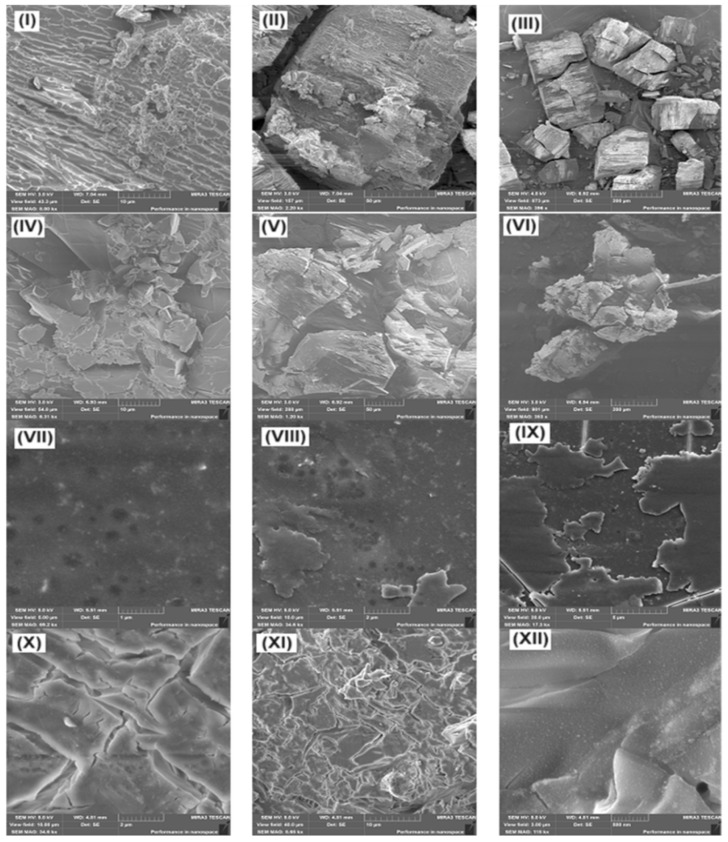
Scanned electron micrographs of samples: (**I**–**III**) Cz-β-CD (1:1); (**IV**–**VI**) CzP-β-CD (1:1); (**VII**–**IX**) Cz-β-CD-AgNPs; (**X**–**XII**) CzP-β-CD-AgNPs.

The change in the morphology of the films of the studied compounds was also analyzed using scanning electron microscopy. Figure 13I–IX shows the micrographs of Cz and CzP and their inclusion complexes before annealing (Cz-β-CD, Cz-β-CD-Ag and CzP-β-CD-Ag). The study of the surface morphology of the inclusion complexes Cz-β-CD and Cz-β-CD-AgNPs showed that these nanocomposites are quite complex structural and morphological organizations. In the SEM photographs of the surface of the inclusion complex samples (Figure 13VII–IX) the diameter of silver particles varies from units to several tens of nanometers. As follows from the data in Figure 13X–XII, the thermal stress arising during heating also causes cracks and deformations in the clathrate material.

In order to fully understand the changes in the morphology of the Cz, CzP, Cz-β-CD, Cz-β-CD-AgNPs and CzP-β-CD-AgNPs complexes during heat treatment in a nitrogen atmosphere, studies were carried out to analyze the evolution of their structure under different temperature conditions (Figures S7–S11). In the SEM images of Cz (Figure S7), low-temperature treatment up to 90 °C (Figure S7I–III) caused virtually no changes. In the images (Figure S7I–III) only minor changes in texture are observed (the characteristics of the crystal structure have changed), but no significant loss of integrity is observed.

At temperatures above 160 °C (Figure S7IV–VI) more pronounced changes can be observed—minor transformations in the morphology of the particles occur. During primary recrystallization of the particles, the clarity of the lines decreases, and the structure becomes more homogeneous (Figure S7IV–VI). When the temperature increases to 250 °C (Figure S7VII–IX) significant changes in the microstructure of the particles are recorded. Recrystallization processes and the beginning of phase transformations are observed. Crystalline phases become more clearly expressed, and new structural forms appear. At a temperature of 315 °C (Figure S7X–XII) an increase in the size of the crystals is observed and new phases begin to form. In this temperature zone, there are risks of the appearance of both homogeneous structures and unevenness in their distribution (Figure S7X–XII). At a temperature of 360 °C (Figure S7XIII–XV) a complete decomposition of a number of initial structures and the formation of new phases occurs. Significant destruction of the crystal lattice is observed, which leads to a change in the appearance of the sample and makes it less homogeneous (Figure S7XIII–XV).

SEM images of cytophate (CzP) (Figure S8I–III) also show a variety of textures, ranging from smooth to rough areas, which may indicate a decrease in the strength of the bonds between molecules and the ability to form macroscopic structures (Figure S8I–III). Upon heating to 250 °C, dissociation of individual particles is observed, which leads to a change in their overall size and distribution pattern. This phenomenon is associated with thermal activity causing the rupture of chemical bonds and is accompanied by decomposition into smaller components (Figure S8VII–IX). Upon prolonged heating to 360 °C, specific damage such as microcracks is formed. These defects significantly affect the mechanical properties of CzP and become clearly visible in SEM images (Figure S8X–XII).

Annealing at 80 °C leads to deformation of the Cz-β-CD inclusion complex (Figure S9). The SEM image shows heterogeneity of the sample surface with a diverse texture, including many microscopic protrusions and pores with a diameter of about 33–55 nm. The convex region of the Cz-β-CD inclusion complex after heat treatment is represented by sintered particles (Figure S9I). Upon closer examination in Figure S9II, it can be seen that these sintered particles contain smaller compacted components, the surface of which is located within 400–700 nm. Empirical observations indicate the presence of slip lines on the grain surface (Figure S9I–III). After heat treatment of the Cz-β-CD inclusion complex to 270 °C, significant changes in the clathrate morphology occurred. The formation of differently oriented groups of slip lines (Figure S9V) and incipient microcracks (Figure S9VI) were revealed on the surface of the Cz-β-CD inclusion complex.

Figure S9VII–IX shows micrographs of Cz-β-CD inclusion complex samples after heat treatment at 300 °C. Heat treatment of the Cz-β-CD inclusion complex results in the formation of numerous groups of slip lines with different orientations on the surface (Figure S9VII) and the occurrence of secondary cracks (Figure S9VIII). Investigation of the microstructure of the Cz-β-CD complex showed that fracture is usually multifocal and propagates in different directions depending on external influences (Figure S9XI,XII). Figure S9XIII–XV shows SEM images of the Cz-β-CD inclusion complex after heat treatment at 450 °C. It can be seen that the initially continuous phase after deformation of the Cz-β-CD inclusion complex is destroyed in the form of islands (Figure S9XIV,XV). SEM images of the surface of the Cz-β-CD-Ag inclusion complex after heat treatment at 90 °C show silver particles up to 365 nm in size (Figure S10I–III). According to the SEM data, the Cz-β-CD-Ag inclusion complex has a predominantly spherical shape (Figure S10I–III). This indicates the presence of silver nanoparticles in the structure of the complex and their changes under the influence of heat. Additional changes in the structure and properties of the Cz-β-CD-Ag inclusion complex were observed after heating the sample at a temperature of 160 °C (Figure S10IV–VI). Most likely, at a temperature of 160 °C, more intense thermal degradation of the complex components occurs, which can affect their stability.

The obtained micrographs (Figure S10IV–VI) show individual metallic silver formations measuring 50–100 nm, presumably with a spherical shape. Heating the Cz-β-CD-Ag inclusion complex to a temperature of 250 °C noticeably changes its morphology. According to the SEM images of the surface of the Cz-β-CD-Ag inclusion complex samples formed at a temperature of 250 °C, rounded particles are present, the average size of which is about 3 nm (Figure S10VII–IX). Upon further heat treatment, particles measuring up to 50 nm and more appear (Figure S10VII–IX). Thus, it can be assumed that the observed spherical aggregates are silver particles, which increase in size upon further heat treatment.

After heat treatment of the Cz-β-CD-Ag inclusion complex at 315 °C, the predominant presence of non-aggregated round particles is observed (Figure S10X–XII). Significant changes in the morphology of the Cz-β-CD-Ag inclusion complex are observed after their heat treatment at 360 °C. As follows from Figure S10XIII,XIV, the inclusion complex contains polydisperse spherical particles (the size of the main fraction is 50–100 nm). The SEM images of the CzP-β-CD-Ag sample (Figure S11I–III) show similar changes in the surface morphology caused by thermal exposure. In particular, microcracks and deformations of the sample structure are visible in Figure S11I–III. When the CzP-β-CD-Ag inclusion complex is heated to 250 °C, an increase in the texture porosity is observed due to thermal decomposition of the organic components (Figure S11IV–VI). This phenomenon can be accompanied by the formation of cracks and deformations in the structure of the nanocomposite material. Under these conditions, agglomeration of Ag nanoparticles and sintering of the matrix are also possible, which could lead to a change in the mechanical properties of the composition (Figure S11VII–IX). As a result of sudden heating caused by the difference in the temperature expansion coefficients of the components of the complex, cracks are formed in its structure. This is clearly visible on the surface of the particles, which also changes the perceived texture (Figure S11X–XII). As follows from the data in Figure S11XIII–XV, silver nanoparticles are clearly visible in the figures, the linear size of which is mainly in the range of 50–60 nm. Note that the shape of the particles does not have a pronounced clarity, which is an expected phenomenon for such objects.

### 2.4. Kinetic Analysis of Thermal Decomposition of Cz, CzP, Cz-β-CD and Cz(CzP)-β-CD-AgNPs

Reactions involving solids, such as the thermal decomposition of Cz, CzP and their inclusion complexes Cz-β-CD and Cz(CzP)-β-CD, as well as their AgNPs-modified derivatives, are classified as topological processes with the reaction zone localized at the interface of the solid reactant and product [41]. Under such conditions, the concentration of the reactant loses its significance, and it is more convenient to use the parameter α, which represents the fraction of the reacted substance at a certain point in time. The initial value of the parameter α is 0 (at the initial point in time), and at the end of the process α reaches the value of 1. A mathematical model of such reactions can be represented using a differential equation with an initial condition reflecting the value of α for reagent A at the start of the reaction (t = 0). In the context of topological processes involved in the thermal decomposition of solids, it is important to take into account the features of reaction localization on the surface of solid phases and the dynamics of changes in the degree of conversion of reagents into products. Almost all methods for calculating kinetic parameters from thermogravimetric data are based on the application of the equation [41]:

(1) $$\frac{d\alpha }{\mathrm{dt}}-\beta \frac{d\alpha }{\mathrm{dT}}-k\left(T\right)f\left(\alpha \right)-\mathrm{Aexp}\left(-\frac{E_{\alpha }}{\mathrm{RT}}\right)f\left(\alpha \right)$$

The interest of scientists [41,42,43,44] in the course of conducting research is attracted by the fact that there is no direct connection between the kinetic characteristics calculated on the basis of isothermal data and the chosen model. At the same time, methods based on non-isothermal approaches demonstrate an inverse relationship [45]. The Friedman method [42,43] is the most common and frequently used isoconversion method. This method is based on the following equation:

(2) $$\ln\left(-\frac{\mathrm{dx}}{\mathrm{dt}}\right)=\mathrm{lnA}+\mathrm{lnf}(x)-E/\mathrm{RT}$$

Many approximations have the general form of a linear equation:

(3) $$\ln\left(\frac{\beta_{i}}{T\beta_{\alpha ,i}}\right)=\mathrm{const}-C\left(\frac{E_{\alpha }}{{\mathrm{RT}}_{\alpha }}\right)$$

where *β_a,i_* and *C* are the parameters that determine the type of temperature integral approximation.

**Figure 14:**
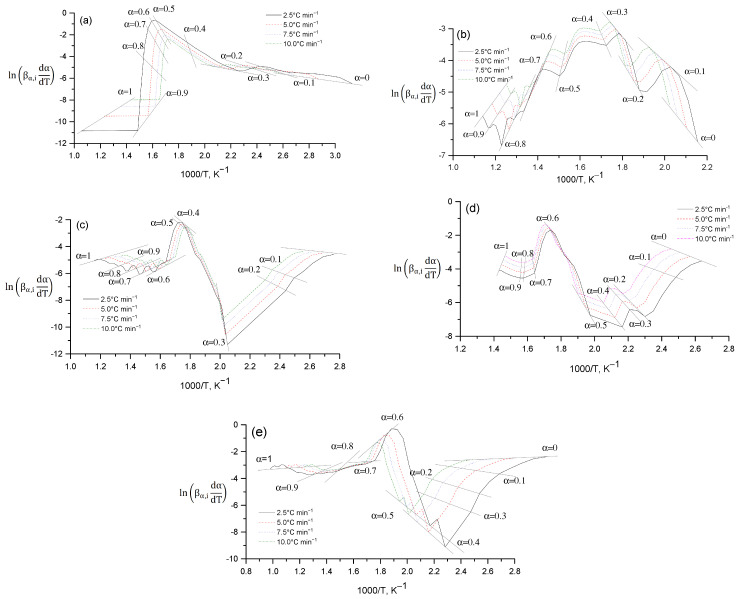
Schematic representation of the Friedman differential method: Cz (**a**); CzP (**b**); Cit-β-CD (**c**); Cit-β-CD-AgNPs (**d**); CzP-β-CD-AgNPs (**e**).

The Friedman method (differential method) can be visually represented through a schematic representation, which includes a detailed description of the steps and processes used to solve differential equations. Figure 14 shows the dependence of $\ln\left(\beta_{\alpha ,i}\frac{d\alpha }{dT_{\alpha }}\right)$ on the reciprocal temperature 1000/T at different heating rates ($\beta_{\alpha ,i}$): 2.5 °C min^−1^, 5.0 °C min^−1^, 7.5 °C min^−1^, 10.0 °C min^−1^. Each curve (Figure 14) corresponds to fixed values of the degree of conversion (α) in the range from 0 to 1. In the initial stages of the process (α = 0.1–0.4), low kinetic activity is observed, which is reflected in the increase in the value of 1000/T. With an increase in the degree of conversion (α = 0.5–0.9), a sharp increase in the value of $\ln\left(\beta_{\alpha ,i}\frac{d\alpha }{dT_{\alpha }}\right)$ occurs, which corresponds to the active stage of decomposition or transformation of the substance. At the end of the process (α = 1) the curves gradually decrease, reflecting the reaction slowdown. An increase in the heating rate leads to a shift of the curve peaks towards higher temperatures. This shift is associated with a thermal delay in the reaction process and reflects the dependence of the kinetic parameters on temperature. The graph (Figure 14) illustrates the application of the isokinetic approach to determining the kinetic parameters of the process, including the activation energy, using the differential Friedman method.

The nonparametric kinetics method, like the Friedman method, allows one to effectively describe the nature of reactions as the process rate increases. However, one of the key features of this approach is the ability to more flexibly determine formal kinetic parameters based on a given set of data (dα/dt, T and α) without the need for a preliminary assumption about the reaction model, which distinguishes it from the classical Friedman method. Let us consider in more detail the mathematical side of the nonparametric kinetics method. The nonparametric kinetics method [46] is based on the construction of a matrix containing information on *k*(*T*) and *f*(*α*). The approach to this matrix can be presented using the singular value decomposition algorithm [47,48].

Below is the kinetic matrix of the NPK method, in which the reaction rate is represented as the product of two independent functions, $k\left(T\right)=\left[\Sigma_{1}v_{1},\Sigma_{1}v_{2},\ldots \Sigma_{1}v_{j}\right]$ and $f\left(\alpha \right)=\left[u_{1},u_{2},\ldots v_{i}\right]$.

(4) $$\mathrm{Kinetic}\;\mathrm{matrix}:\;M=\left\{m_{\mathrm{ij}}\right\}=\left\{\begin{array}{cccc} r\left(\alpha_{1},T_{1}\right) & r\left(\alpha_{1},T_{2}\right) & \ldots & r\left(\alpha_{1},T_{j}\right)\\ r\left(\alpha_{2},T_{1}\right) & r\left(\alpha_{2},T_{2}\right) & \ldots & r\left(\alpha_{2},T_{j}\right)\\ \ldots & \ldots & \ldots & \ldots \\ r\left(\alpha_{i},T_{1}\right) & r\left(\alpha_{i},T_{2}\right) & \ldots & r\left(\alpha_{i},T_{j}\right) \end{array}\right\}$$

**Figure 15:**
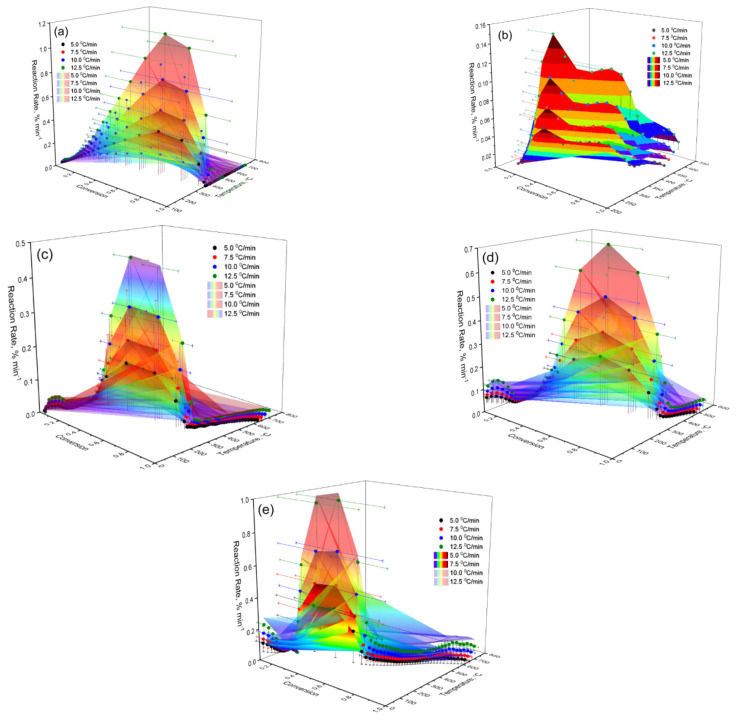
Three-dimensional coordinate system: (**a**) Cz; (**b**) CzP; (**c**) Cit-β-CD; (**d**) Cz-β-CD-AgNPs; (**e**) CzP-β-CD-AgNPs.

The experimental data on the reaction rate were obtained by calculating according to Equation (4) and are presented in a three-dimensional coordinate system. As can be seen from Figure 15a, the maximum reaction rate is achieved at an average degree of conversion (α = 0.5–0.7). Under these conditions, with an increase in the heating rate, a shift in its peaks to higher temperatures is observed. These data indicate the energy dependence of the process and confirm its multistage nature. The spatial distribution of the reaction rate values on the graph allows visualizing the influence of thermal conditions on the kinetic behavior of the system under study.

After applying the singular value decomposition (SVD) algorithm, the matrix M is a vector S with two significant values. In this case, the matrix M can be expressed as a sum:

*M* = *M*_1_ + *M*_2_ = *u*_1_*v*_1_^*T*^ + *u*_2_*v*_2_^*T*^ (5)

This suggests that there are two underlying processes at the decomposition stage, and the difference between them can be determined by the values of the explained variance. The vectors were compared with the Šesták–Berggren equation and the Arrhenius equation, respectively [46,49].

*f*(*a*) = *α*^*m*^(1 − *α*)^*n*^ (6)

**Figure 16:**
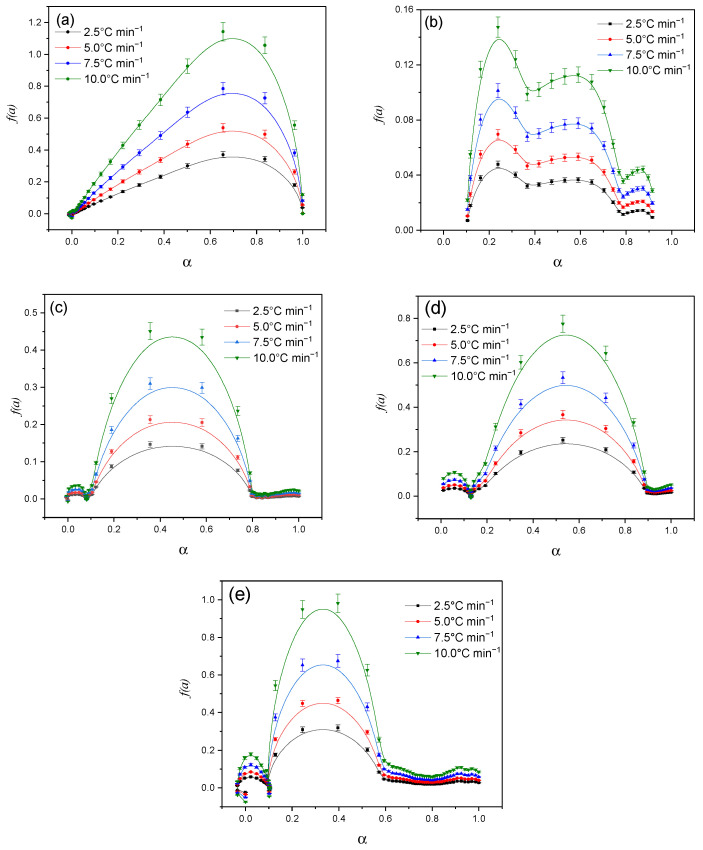
Graphical solution to the Šesták and Berggren model: Cz (**a**); CzP (**b**); Cz-β-CD (**c**); Cz-β-CD-AgNPs (**d**); CzP-β-CD-AgNPs (**e**).

Figure 16 shows the dependence of the reaction rate (*dα/dt*) on the degree of conversion (α) for different heating rates (2.5 °C min^−1^, 5.0 °C min^−1^, 7.5 °C min^−1^, 10.0 °C min^−1^). The obtained data demonstrate a characteristic reaction profile, in which the reaction rate increases with increasing degree of conversion up to a certain maximum (0.4–0.60) and then decreases as the process is completed (α→1). With increasing heating rate, the peak of the reaction rate shifts to the region of higher α values, which is associated with a change in the energy barriers of the process.

**Figure 17:**
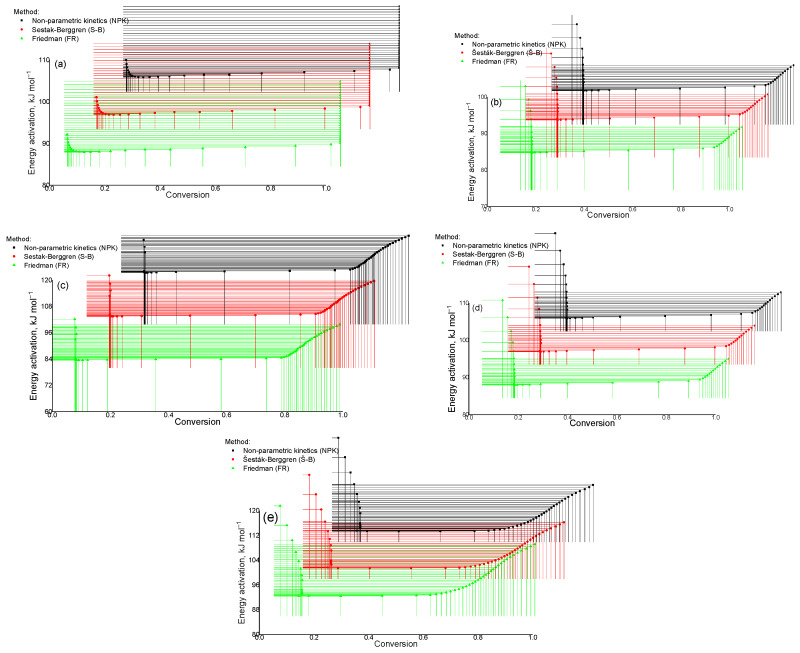
Activation energy (E) indices determined using the methods of nonparametric kinetics (NPK), Šesták–Berggren (SB) and Friedman (FR): Cz (**a**); CzP (**b**); Cz-β-CD (**c**); Cz-β-CD-AgNPs (**d**); CzP-β-CD-AgNPs (**e**).

As follows from the data in Figure 17a, with a decrease in the parameter α = 0.1 the process becomes slower, whereas its increase leads to an acceleration of the thermal destruction reaction. These results emphasize the importance of monitoring this parameter for the effective regulation of the kinetics of processes in the studied system of substances. The scheme presented in Figure 17b shows that with an increase in the activation energy (*α* = 0.1) the decomposition process of the Cz-β-CD complex slows down, whereas with a decrease in this parameter (*α* = 0.2 to *α* = 0.8) the process proceeds more quickly. Similar behavior is observed when analyzing the thermal characteristics of the Cz(CzP)-β-CD-AgNP composites. This indicates a significant effect of changes in the activation energy on the decomposition rate of these complexes.

Table 1 presents the kinetic parameters of thermal decomposition of various compounds including β-CD, Cz, CzP and their complexes with silver nanoparticles (Cz(CzP)-β-CD-AgNPs). The calculated parameters include the activation energy (Ea), pre-exponential factor (A) and the values of α^m^(1 − α)^n^ parameters for the Šesták–Berggren model. For β-CD, the activation energy is 83.94–84.60 kJ/mol, while for Cz this parameter is 89.05 kJ/mol. The highest value of ${\overset{-}{E}}_{a}$ is observed for the CzP-β-CD-AgNPs complex and is 93.03–94.32 kJ/mol, which indicates increased thermal stability of this compound. A similar trend is observed for the pre-exponential factor (A), which is significantly higher for complexes with silver nanoparticles, such as CzP-β-CD-AgNPs (A = 2.89 × 10^18^–1.05 × 10^12^). The parameters of the Šesták–Berggren model (m and n) also show differences for different compounds. As can be seen from Table 1, for β-CD, the values of the parameters m and n are 0.47 and 0.53, respectively, while for CzP-β-CD-AgNPs they reach 0.66 and 1.34. This indicates differences in the mechanism of thermal decomposition of the studied materials. The obtained data emphasize the influence of the composition of the complexes and the presence of silver nanoparticles on the kinetic parameters of thermal decomposition, demonstrating a significant role of energy and structural factors in the stability of the compounds.

## 3. Materials and Methods

### 3.1. Preparation of β-CD-Cz and β-CD-CzP Inclusion Complexes

The following reagents were used: cytisine base (Cz), white crystals, C_11_H_14_N_2_O, molar mass 190.24 g/mol, mp. 152–153 °C, “analytical grade” (Aldrich, Tashkent, Uzbekistan); β-CD (99%) (Fluka, mp 270–290 °C with decomp.). Silver nitrate (AgNO_3_) and hydroxide NaOH were purchased from Sinopharm Chemical Reagent (Shanghai, China). O,O-dimethyl-N-cytisinylamidophosphate (CzP) was prepared according to the procedure [50]. The inclusion complexes of Cz-β-CD (1:1) (mp. 290–310 °C with dec.) and CzP-β-CD (1:1) (mp. 280–300 °C with dec.) were obtained by coprecipitation in an aqueous-alcoholic medium [33]. The yields of clathrates, encapsulation efficiency and loading of Cz(CzP)-AgNPs were analyzed using a UV–visible spectrophotometer (Evolution 220, Thermo Fisher Scientific, Waltham, MA, USA) according to the method described in [51]. All experiments with Cz-β-CD (1:1) and CzP-β-CD (1:1) were carried out at a temperature of (25 ± 0.5) °C and each experiment was repeated three times. The yields of clathrates (Y, %), incorporation efficiencies (IE, %) and guest molecule loading (DL, %) were calculated using the following equations [52,53]:

Y, % = (W_d_/(W_CD_ + W_Cz(CzP)_)) × 100 (7)

IE, % = (W_in_/W_Cz(CzP)_) × 100 (8)

DL, % = (W_in_/W_d_) × 100 (9)

where W_d_ is the mass of the inclusion complex, W_Ct_ and W_CD_ are the masses of Cz and β-CD added to the inclusion complex system, W_in_ is the weight of Cz(CzP) in the inclusion complex.

The following results were obtained for Cz(CzP)-β-CD (1:1). Yields of inclusion complexes (Y, %) 65.43 ± 0.35 (62.25 ± 0.42); inclusion efficiency (IE, %) 78.11 ± 0.56 (71.03 ± 0.23); loading of Cz(CzP) molecules (DL, %) 10.36 ± 0.22 (8.75 ± 0.11).

### 3.2. Preparation of Cz-β-CD-AgNPs and CzP-β-CD-AgNPs

β-CD-AgNPs were synthesized by an in situ reduction method according to the described method [34]. A total of 5 mL of β-CD solution (0.01 M) was added to 32.5 mL of water, to which NH_4_OH (10%) was gradually added under stirring until pH value was 9. After heating the solution to 60 °C, AgNO_3_ (0.05 M) was added dropwise, and further reaction was carried out for 2 h at 70 °C to obtain a yellow-brown solution of β-CD-AgNPs. With further reaction, the color of the solution changed to intense yellow-brown. The resulting solutions of β-CD-AgNPs were then used to encapsulate Cz and CzP molecules. The Cz-β-CD-AgNPs complex was obtained by slowly adding 5 mL of Cz solution (0.01 m) to the β-CD-AgNPs solution and stirring for 24 h at 25 °C. β-CD-CzP-AgNPs was obtained in a similar way. The synthesis scheme of β-CD-Cz(CzP)-AgNPs is shown in Figure 1. The absorption spectra of Cz-β-CD-AgNPs (**1**) and CzP-β-CD-AgNPs (**2**) in the UV range at 423.30 and 433.15 nm, respectively, indicated that the obtained nanoparticles have absorption peaks characteristic of spherical nanoparticles [34,35].

### 3.3. Defining Characteristics

Absorption spectra in the ultraviolet range were recorded using an N60 Implen UV-visible spectrophotometer (München, Germany). Samples of substances for analyzing spectra in the ultraviolet range were prepared by mixing 1 mL of solution with 10 mL of water. ^1^H, ^13^C NMR and COSY, HMQC and HMBC spectra were obtained using a Bruker Avance 600 M NMR instrument using D_2_O as a solvent. FT-IR spectrum was measured on a Nicolet 6700 Fourier external spectrometer (Nicolet, ON, Canada). The samples were mixed with KBr and pressed into pellets. A total of 32 scans were acquired, ranging from 4000 to 400 cm^−1^ at a resolution of 4 cm^−1^. XRD pattern was measured on an XD6 X-ray diffractometer at 40 kV and 30 mA with a scanning speed of 5° per min and a scanning range of 20–90° by using CuKa radiation (l = 0.1546 nm). The shape, size and distribution of AgNPs were investigated using TEM. The TEM samples were prepared by placing a few drops of as-prepared sol on the surface of the copper mesh covered with a carbon support film and dried at room temperature. The particle size distribution was obtained from the TEM image with the ImageJ sofware 1.45. The surface morphology inclusion complexes (clathrates) samples were studied using a scanning electron microscope (SEM) from Tescon Mira 3 LMN (Brno, Czech Republic). The samples were attached to the conductive adhesive surface and observed at an accelerating voltage of 15 kV.

## 4. Conclusions

The alkaloid cytisine and its O,O-dimethyl phosphate derivative have attracted attention due to their antiviral properties, which inspire the development of methods for their reliable biomedical application. In this study, the synthesis and properties of β-cyclodextrin-functionalized silver nanoparticles and their loading with a drug component are considered. β-Cyclodextrin was used as a reducing agent and stabilizer in the preparation of silver nanoparticles. The use of β-CD-AgNPs in loading molecules of the alkaloid cytisine and its O,O-dimethyl phosphate derivative, which have pronounced antiviral properties, was studied. The obtained kinetic data allow us to predict the stability of their Cz(CzP)-β-CD complexes with silver nanoparticles during long-term storage, as well as to find optimal conditions for their formation.

## Figures and Tables

**Table 1:** Kinetic parameters of the thermal destruction process of β-CD, Cz, CzP and their inclusion complexes Cz-β-CD and Cz(CzP)-β-CD modified with AgNP nanoparticles.

Sample	${\bar{E}}_{\mathrm{NPK}}$, kJmol^−1^	$\bar{A}$, c^−1^	Šesták–Berggren		${\bar{E}}_{S-B}$, kJmol^−1^	$\bar{A}$, c^−1^	${\bar{E}}_{FR}$, kJmol^−1^	$\bar{A}$, c^−1^
			***α^m^(*** **1 − *α*** **)** ***^n^***					
			m	n				
β-CD	83.94	1.56 × 10^15^	0.47	0.53	84.60	4.01 × 10^16^	83.41	2.72 × 10^15^
Cz	89.05	1.46 × 10^5^	0.65	0.34	89.05	2.02 × 10^6^	89.05	1.46 × 10^5^
CzP	90.12	1.54 × 10^18^	0.51	0.75	91.09	2.05 × 10^19^	90.01	2.14 × 10^18^
Cz-β-CD	93.73	1.25 × 10^18^	0.35	0.64	93.59	1.00 × 10^19^	91.23	1.76 × 10^11^
Cz-β-CD-AgNPs	85.62	2.04 × 10^10^	0.53	0.47	85.09	7.82 × 10^10^	85.62	2.10 × 10^10^
CzP-β-CD-AgNPs	93.03	2.89 × 10^18^	0.66	1.34	94.32	1.05 × 10^12^	90.94	1.01 × 10^12^

## Data Availability

The data that support the findings of this study are available within the article and the Supplementary Materials. Further data are available from the corresponding author upon reasonable request.

## Supplementary Materials

The following supporting information can be downloaded at https://www.mdpi.com/article/10.3390/molecules30061337/s1, Figure S1. 1H (a), 13C (b) NMR spectra of β-CD-AgNPs (D2O); Figure S2. COSY (a), HMQC (b), HMBC (c) spectra of β-CD-AgNPs, (D2O); Figure S3. 1H (a) and 13C (b) NMR spectra of Cz-β-CD-AgNPs (D2O); Figure S4. COSY (a), HMQC (b), HMBC (c) spectra of Cz-β-CD-AgNPs, (D2O); Figure S5. 1H (a) and 13C (b) NMR spectra of CzP-β-CD-AgNPs (D2O); Figure S6. COSY (a), HMBC (b) spectra of CzP-β-CD-AgNPs (D2O); Figure S7. SEM image of Cz sample after heat treatment at 90 °C (I, II, III), 160 °C (IV, V, VI), 250 °C (VII, VIII, IX), 315 °C (X, XI, XII), 360 °C (XIII, XIV, XV); Figure S8. SEM image of CzP sample after heat treatment at 90 °C (I, II, III), 160 °C (IV, V, VI), 250 °C (VII, VIII, IX), and 360 °C (X, XI, XII); Figure S9. SEM image of the Cz-β-CD inclusion complex (1:1) after heat treatment at 80 °C (I, II, III), 270 °C (IV, V, VI), 300 °C (VII, VIII, IX), 350 °C (X, XI, XII), 450 °C (XIII, XIV, XV); Figure S10. SEM image of a sample of the Cz-β-CD-Ag inclusion complex after heat treatment at 90 °C (I, II, III), 160 °C (IV, V, VI), 250 °C (VII, VIII, IX), 315 °C (X, XI, XII), 360 °C (XIII, XIV, XV); Figure S11. SEM image of a sample of the CzP-β-CD-Ag inclusion complex after heat treatment at 90 °C I, II, III), 160 °C (IV, V, VI), 250 °C (VII, VIII, IX), 315 °C (X, XI, XII), 360 °C (XIII, XIV, XV).
