# Supplementary material for: Synthesis of β-Cyclodextrin-Functionalized Silver Nanoparticles and Their Application for Loading Cytisine and Its Phosphorus Derivative
Source: Molecules. 2025 Mar 17;30(6):1337. doi: 10.3390/molecules30061337 (PMC11944286; doi:10.3390/molecules30061337)
Supplement: Supplementary file 1 [file molecules-30-01337-s001.zip › molecules-3498116-supplementary.pdf]

Serik D. Fazylov[1], Oralgazy A. Nurkenov[*,1,2], Zhangeldy S. Nurmaganbetov[1], Akmaral Zh. Sarsenbekova[3], Ryszhan Ye. Bakirova[*,4], Olzhas T. Seilkhanov[5], Alexandr K. Sviderskiy[6], Ardak K. Syzdykov[1,2], Anel Zh. Mendibayeva[1,2]

Synthesis and properties of β-cyclodextrin functionalized silver nanoparticles and their filling with cytisine and its phosphorous derivative

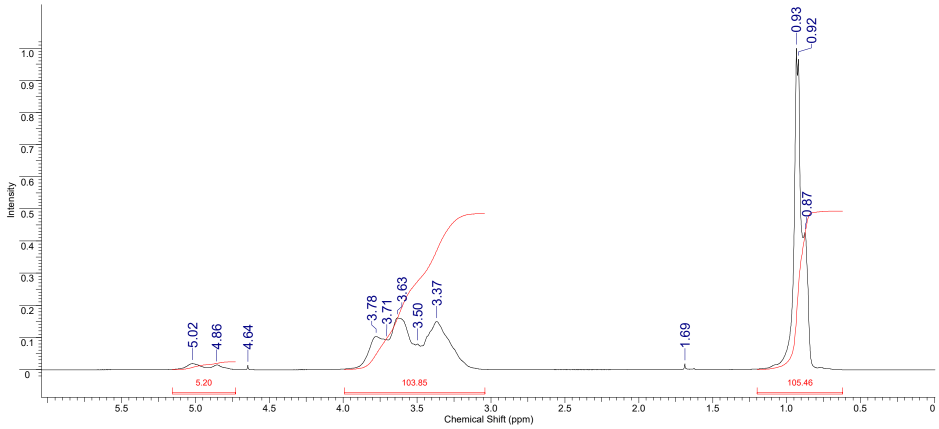

No.	(ppm)	(Hz)	Height
1	0.87	349.4	0.4267
2	0.92	367.2	0.9657
3	0.93	373.2	1.0000
4	1.69	675.0	0.0174
5	3.37	1346.3	0.1493
6	3.50	1397.6	0.0795
7	3.63	1452.1	0.1608
8	3.71	1481.4	0.0925
9	3.78	1510.3	0.1029
10	4.64	1856.9	0.0130
11	4.86	1941.7	0.0139
12	5.02	2006.7	0.0183

No.	(ppm)	Value	Absolute Value
1	[4.73 .. 5.16]	5.200	5.47764e+0
2	[3.04 .. 3.99]	103.846	1.09391e+2
3	[0.62 .. 1.20]	105.463	1.11094e+2

(a)

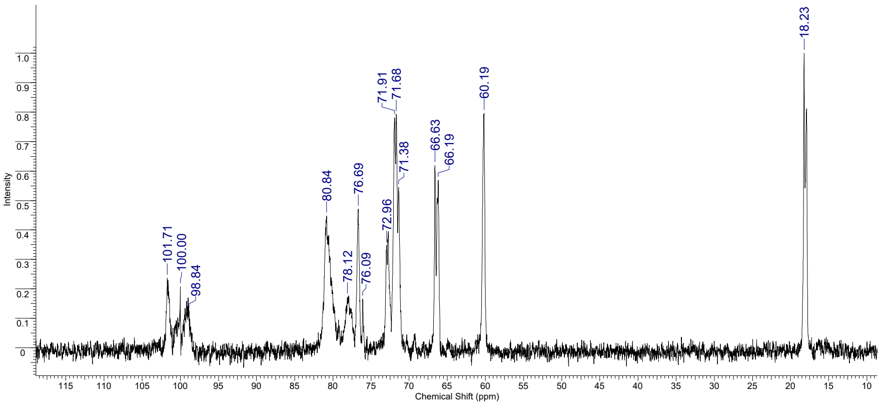

No.	(ppm)	(Hz)	Height
1	18.23	1832.7	1.0000
2	60.19	6050.9	0.7959
3	66.19	6653.8	0.5692
4	66.63	6697.9	0.6187
5	71.38	7175.2	0.5437
6	71.68	7205.9	0.7920
7	71.91	7228.9	0.7810
8	72.96	7334.3	0.3479

No.	(ppm)	(Hz)	Height
9	76.09	7648.7	0.1657
10	76.69	7709.1	0.4712
11	78.12	7852.8	0.1723
12	80.84	8126.0	0.4477
13	98.84	9935.6	0.1255
14	100.00	10052.5	0.2080
15	101.71	10224.1	0.2363

(b)

Figure S1. ^{1}H(a) and ^{13}C(b) NMR spectra of β-CD-AgNPs (D_2O)

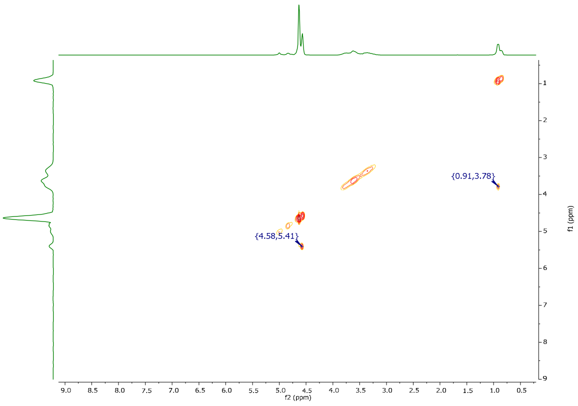

(a)

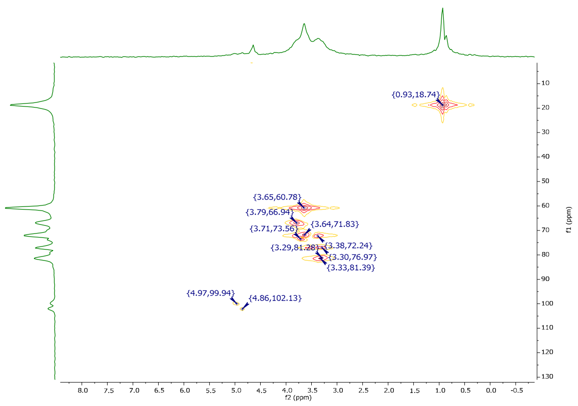

(b)

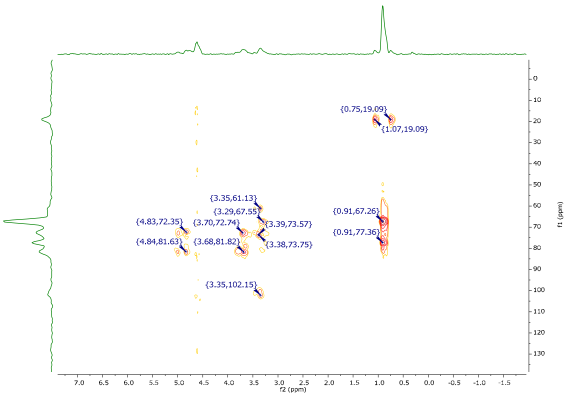

(c)

Figure S2. COSY (a), HMQC (b), HMBC (c) spectra of β-CD-AgNPs (D_2O)

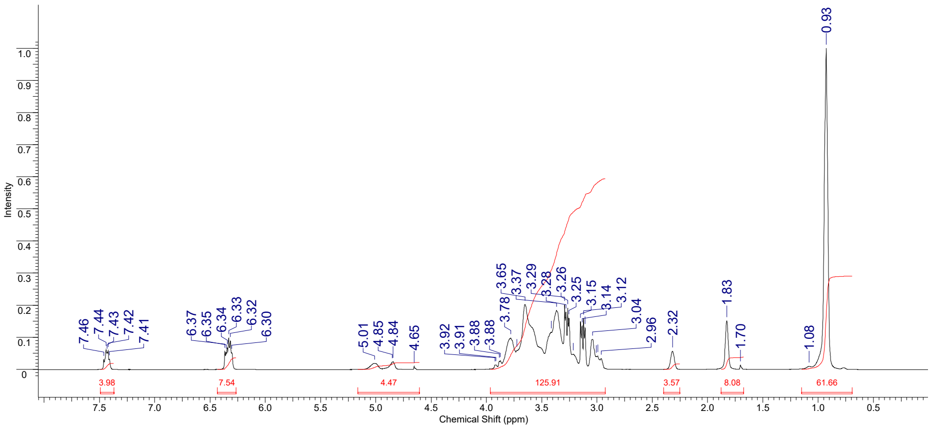

No.	(ppm)	(Hz)	Height	No.	(ppm)	(Hz)	Height	No.	(ppm)	(Hz)	Height
1	0.93	370.9	1.0000	15	3.25	1300.5	0.1600	29	4.84	1936.2	0.0240
2	1.08	432.3	0.0097	16	3.26	1305.1	0.1913	30	4.85	1939.4	0.0231
3	1.70	681.4	0.0137	17	3.28	1311.5	0.1780	31	5.01	2003.5	0.0185
4	1.83	729.9	0.1514	18	3.29	1316.6	0.2012	32	6.30	2519.6	0.0517
5	2.32	926.4	0.0562	19	3.37	1346.3	0.1836	33	6.32	2525.1	0.0890
6	2.96	1184.2	0.0343	20	3.41	1365.1	0.1157	34	6.33	2531.0	0.0977
7	2.99	1197.0	0.0417	21	3.65	1459.9	0.2025	35	6.34	2536.1	0.0676
8	3.01	1201.6	0.0400	22	3.72	1488.8	0.0590	36	6.35	2540.2	0.0532
9	3.04	1215.4	0.0938	23	3.78	1511.2	0.0983	37	6.37	2544.8	0.0552
10	3.11	1242.4	0.1296	24	3.88	1549.2	0.0269	38	7.41	2962.4	0.0319
11	3.12	1247.9	0.1592	25	3.88	1552.9	0.0251	39	7.42	2967.0	0.0581
12	3.14	1253.4	0.1351	26	3.91	1564.8	0.0143	40	7.43	2971.1	0.0531
13	3.15	1258.9	0.1494	27	3.92	1568.4	0.0136	41	7.44	2973.9	0.0606
14	3.22	1285.4	0.0475	28	4.65	1860.6	0.0096	42	7.46	2982.6	0.0309

No.	(ppm)	Value	Absolute Value
1	[2.25 .. 2.40]	3.569	3.72833e+0
2	[7.37 .. 7.49]	3.981	4.15885e+0
3	[4.61 .. 5.16]	4.470	4.66914e+0
4	[6.26 .. 6.43]	7.542	7.87894e+0
5	[1.68 .. 1.88]	8.081	8.44225e+0
6	[0.69 .. 1.15]	61.656	6.44083e+1
7	[2.92 .. 3.96]	125.911	1.31532e+2

(a)

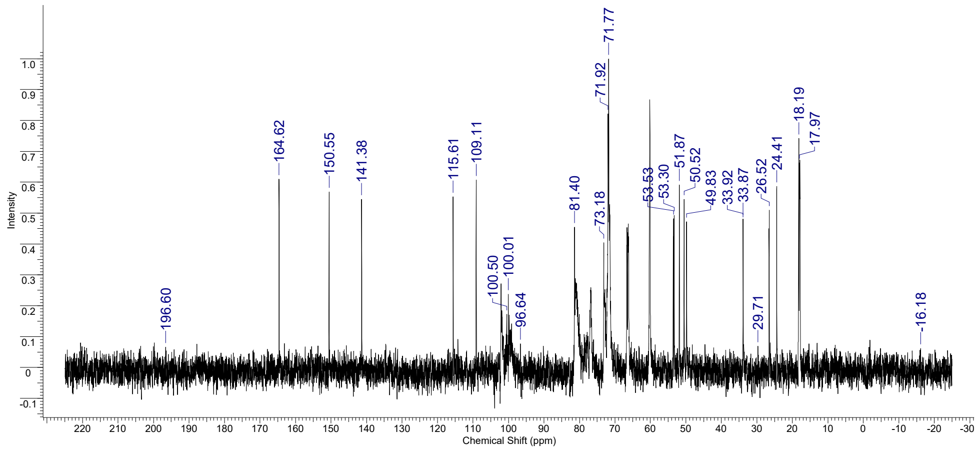

No.	(ppm)	(Hz)	Height	No.	(ppm)	(Hz)	Height	No.	(ppm)	(Hz)	Height
1	-16.18	-1626.4	0.0621	11	50.52	5078.1	0.5458	21	96.64	9715.2	0.0775
2	17.97	1806.8	0.6634	12	51.87	5214.2	0.5915	22	100.01	10053.5	0.2380
3	18.19	1828.9	0.7423	13	53.30	5358.0	0.4933	23	100.50	10102.4	0.1730
4	24.41	2453.8	0.5863	14	53.36	5363.7	0.3950	24	109.11	10967.9	0.6073
5	26.52	2665.6	0.5095	15	53.53	5381.0	0.4819	25	115.61	11621.5	0.5535
6	26.56	2670.4	0.4495	16	53.60	5387.7	0.4248	26	141.38	14212.3	0.5455
7	29.71	2986.7	0.0697	17	71.77	7214.5	1.0000	27	150.55	15134.3	0.5687
8	33.87	3404.6	0.4813	18	71.92	7229.8	0.8217	28	164.62	16548.1	0.6105
9	33.92	3409.4	0.4739	19	73.18	7356.4	0.4051	29	196.60	19763.7	0.0664
10	49.83	5009.1	0.4730	20	81.40	8182.6	0.4547				

(b)

Figure S3. ^{1}H(a) and ^{13}C (b) NMR spectra of Cz-β-CD-AgNPs (D_2O)

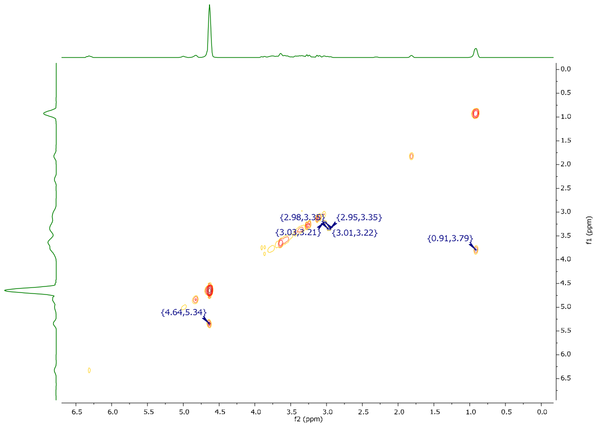

(a)

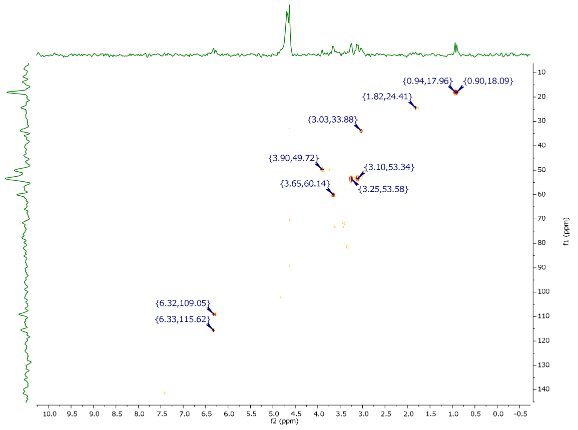

(b)

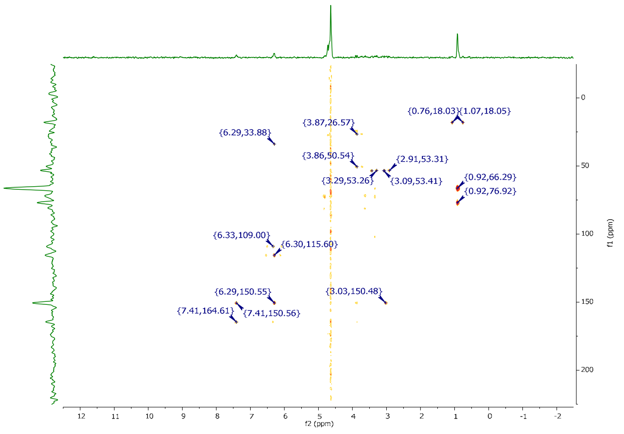

(c)

Figure S4. COSY (a), HMQC (b), HMBC (c) spectra of Cz-β-CD-AgNPs (D_2O)

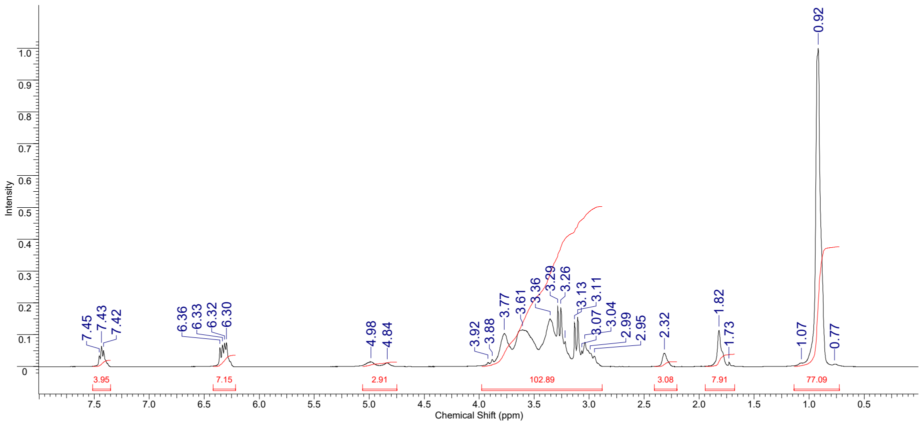

No.	(ppm)	(Hz)	Height
1	0.77	307.3	0.0076
2	0.92	366.8	1.0000
3	1.07	428.2	0.0110
4	1.73	691.0	0.0139
5	1.82	727.7	0.1136
6	2.32	925.9	0.0419
7	2.95	1180.6	0.0327
8	2.99	1195.2	0.0419
9	3.04	1214.9	0.0755
10	3.07	1225.4	0.0485

No.	(ppm)	(Hz)	Height
11	3.11	1241.5	0.1552
12	3.13	1252.5	0.1373
13	3.22	1286.8	0.0786
14	3.26	1302.4	0.1856
15	3.29	1313.8	0.1880
16	3.36	1341.3	0.1493
17	3.61	1441.6	0.1151
18	3.77	1508.0	0.1037
19	3.88	1552.0	0.0221
20	3.92	1567.1	0.0126

No.	(ppm)	(Hz)	Height
21	4.84	1933.9	0.0110
22	4.98	1992.5	0.0144
23	6.30	2517.3	0.0738
24	6.32	2524.6	0.0727
25	6.33	2532.4	0.0679
26	6.36	2541.6	0.0596
27	7.42	2964.7	0.0507
28	7.43	2971.6	0.0635
29	7.45	2980.3	0.0328

No.	(ppm)	Value	Absolute Value
1	[4.75 .. 5.06]	2.911	3.45041e+0
2	[2.20 .. 2.41]	3.081	3.65096e+0
3	[7.35 .. 7.52]	3.946	4.67716e+0
4	[6.21 .. 6.42]	7.153	8.47740e+0
5	[1.68 .. 1.95]	7.914	9.37884e+0
6	[0.72 .. 1.14]	77.092	9.13660e+1
7	[2.88 .. 3.98]	102.888	1.21939e+2

(a)

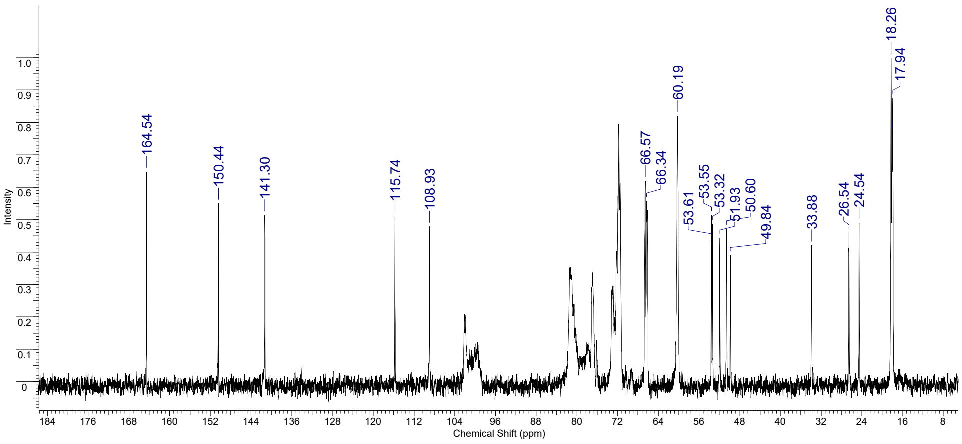

No.	(ppm)	(Hz)	Height
1	17.94	1803.0	0.8751
2	18.07	1816.4	0.7664
3	18.26	1835.6	1.0000
4	24.54	2467.2	0.4893
5	26.54	2667.5	0.4603
6	33.88	3405.6	0.4210
7	33.92	3409.4	0.4062
8	49.84	5010.0	0.3908
9	50.60	5086.7	0.4723
10	51.93	5219.9	0.4435
11	53.32	5359.9	0.4866

No.	(ppm)	(Hz)	Height
12	53.38	5365.6	0.4442
13	53.55	5382.9	0.5150
14	53.61	5389.6	0.4329
15	60.19	6050.9	0.8196
16	66.34	6669.1	0.5583
17	66.57	6692.1	0.6186
18	108.93	10950.6	0.4784
19	115.74	11635.0	0.5074
20	141.30	14204.6	0.5136
21	150.44	15122.8	0.5496
22	164.54	16540.4	0.6478

(b)

Figure S5. ^{1}H(a) and ^{13}C (b) NMR spectra of CzP- β-CD-AgNPs (D_2O)

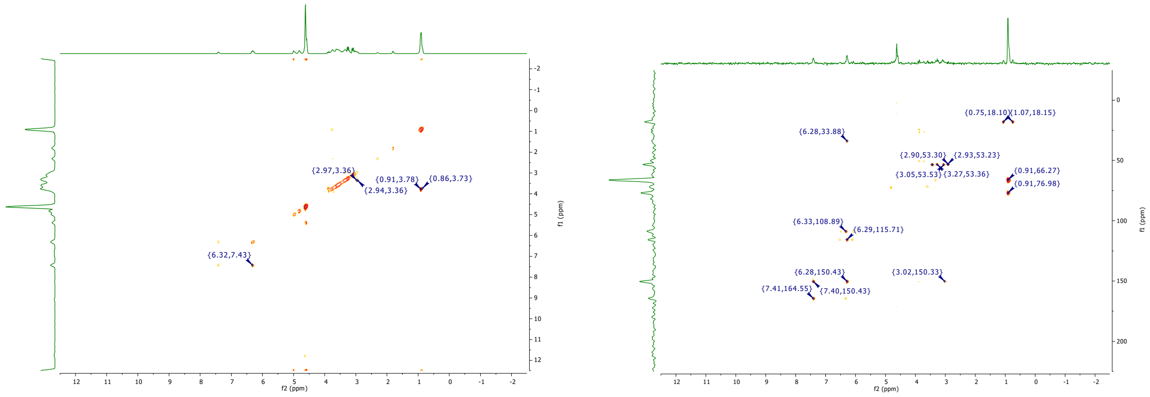

(a) (b)

Figure S6. COSY (a) and HMBC (b) spectra of CzP- β-CD-AgNPs (D_2O)

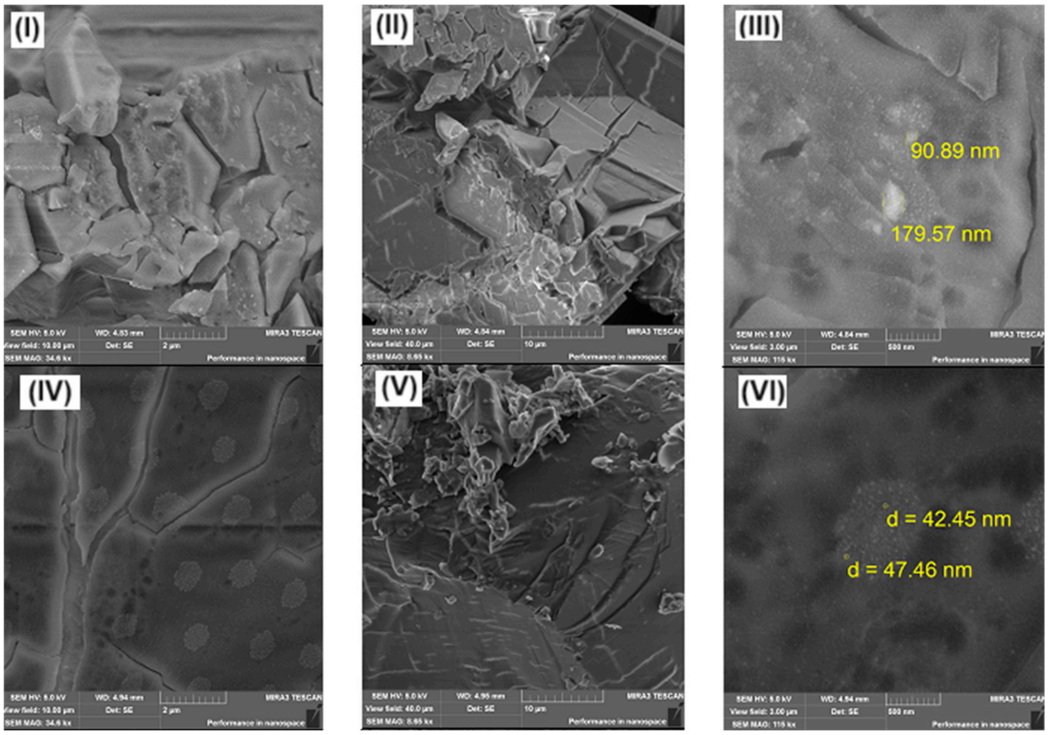

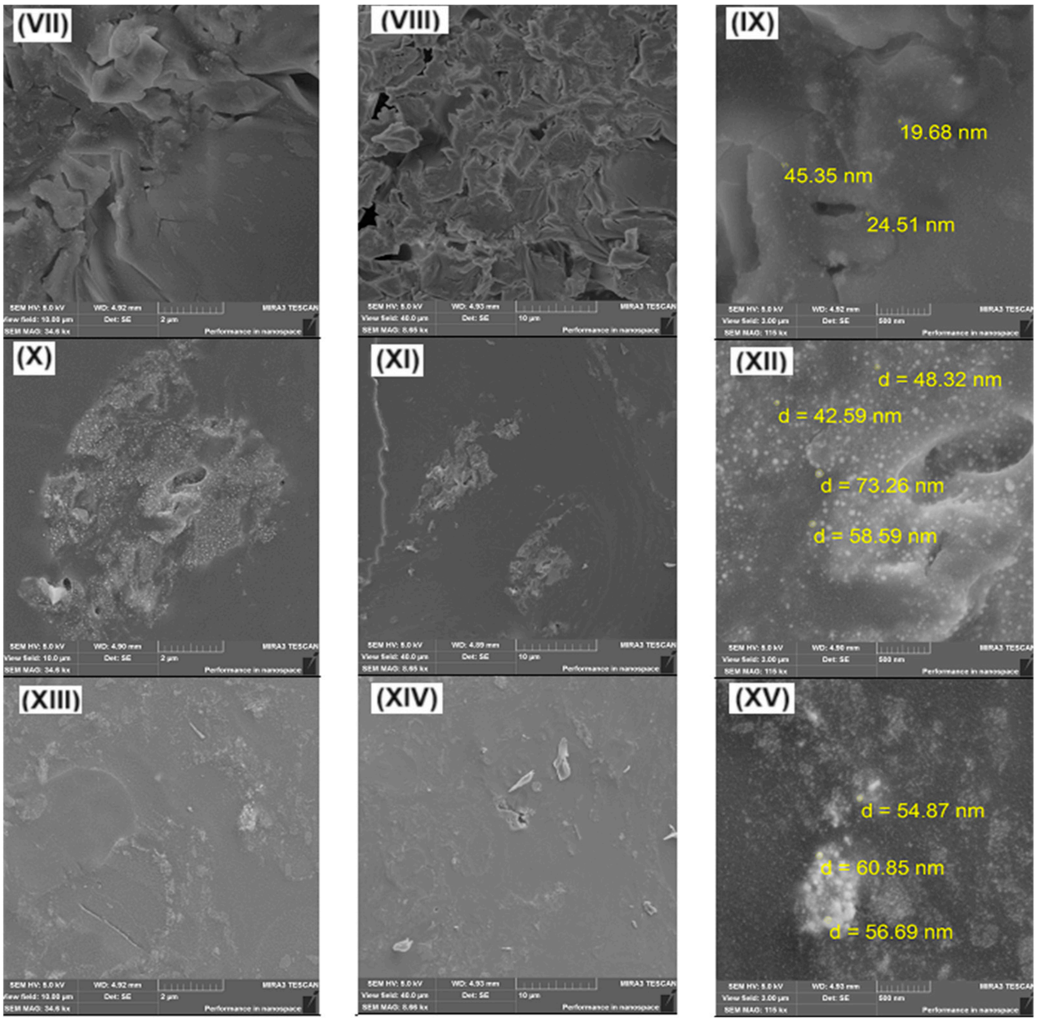

Figure S7. SEM image of Cz sample after heat treatment at 90°C (I, II, III), 160°C (IV, V, VI), 250°C (VII, VIII, IX), 315°C (X, XI, XII), 360°C (XIII, XIV, XV)

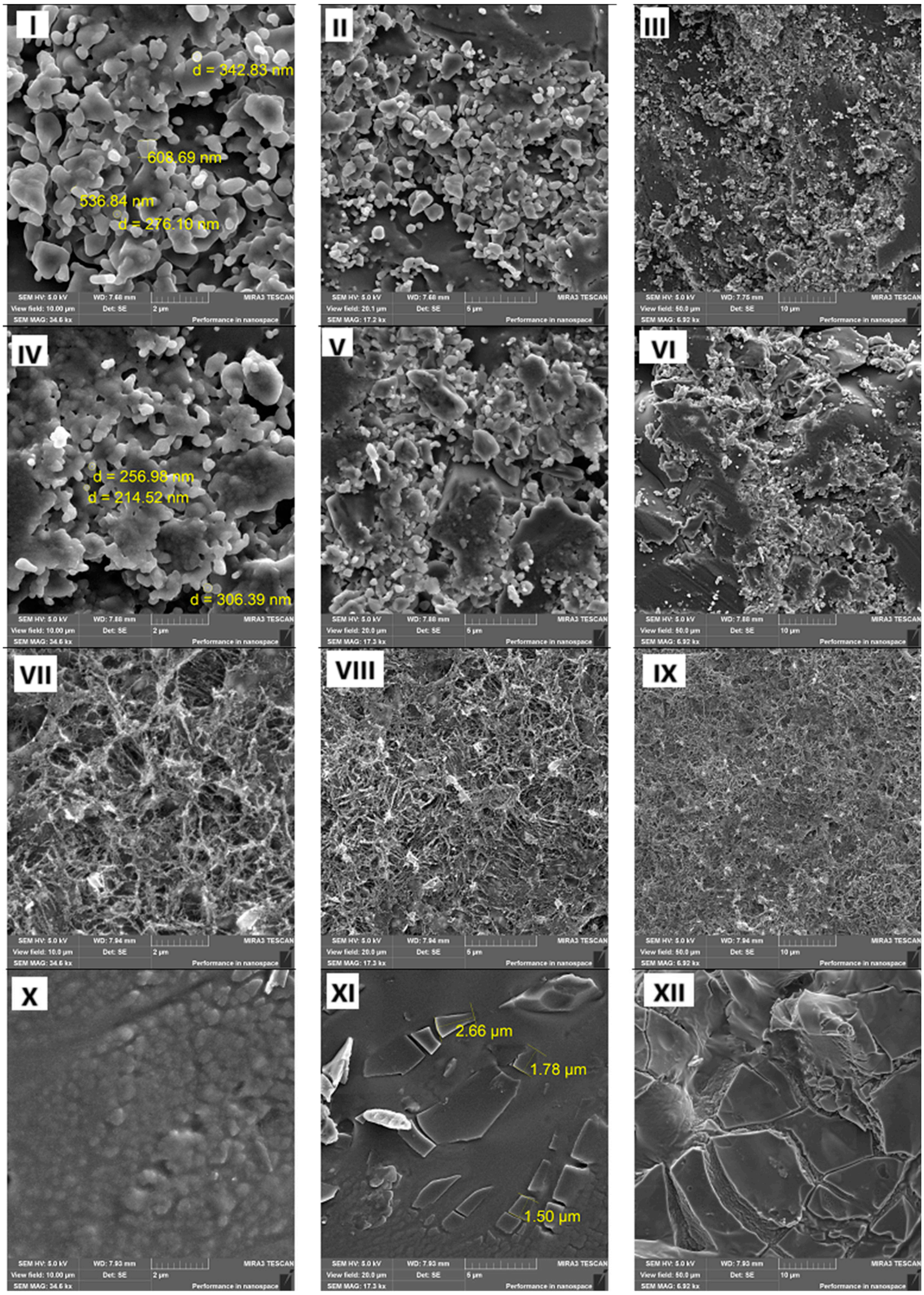

Figure S8. SEM image of CzP sample after heat treatment at 90°C (I, II, III), 160°C (IV, V, VI), 250°C (VII, VIII, IX), and 360°C (X, XI, XII)

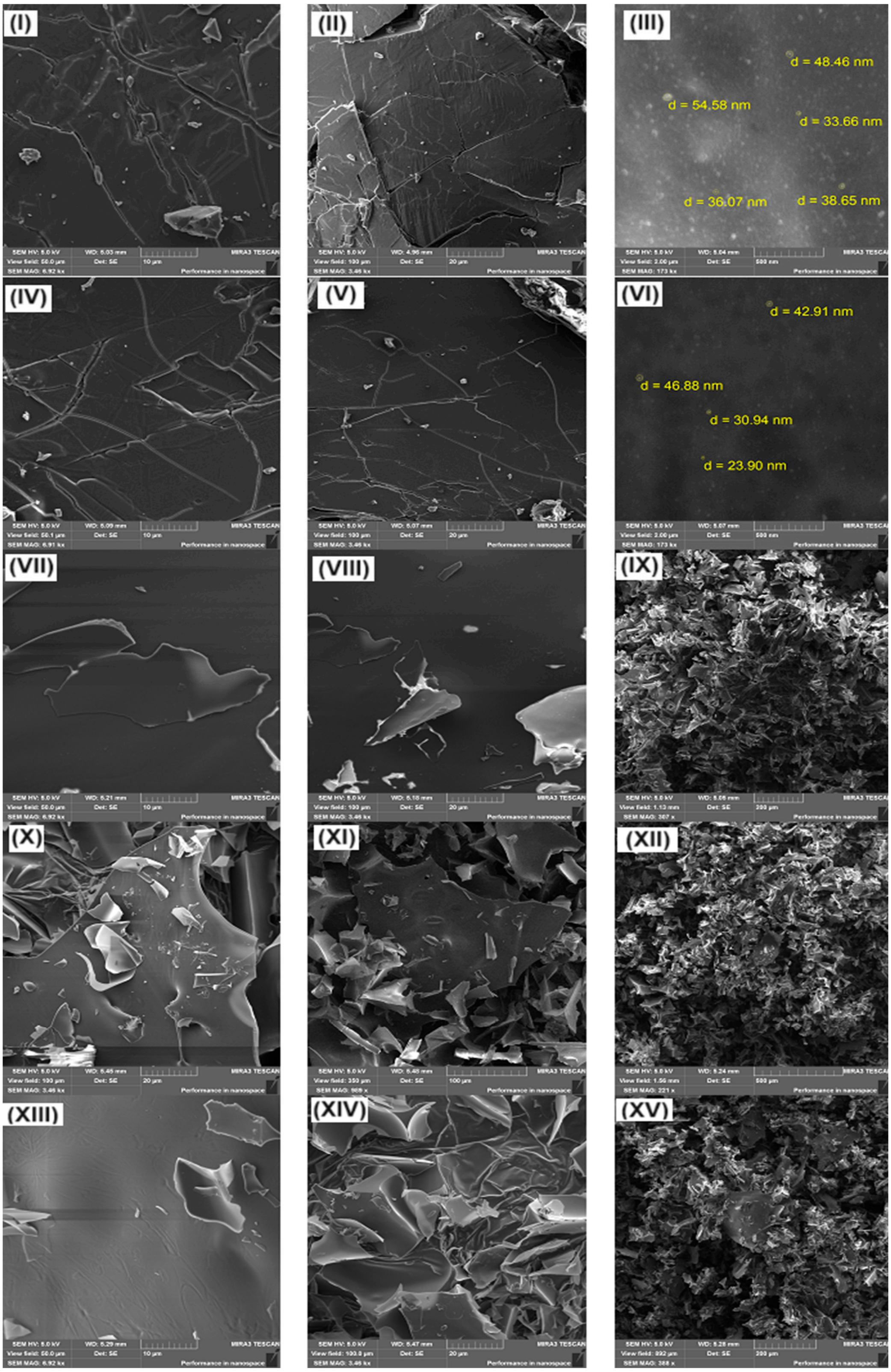

Figure S9. SEM image of the Cz-β-CD inclusion complex (1:1) after heat treatment at 80°C (I, II, III), 270°C (IV, V, VI), 300°C (VII, VIII, IX), 350°C (X, XI, XII), 450°C (XIII, XIV, XV)

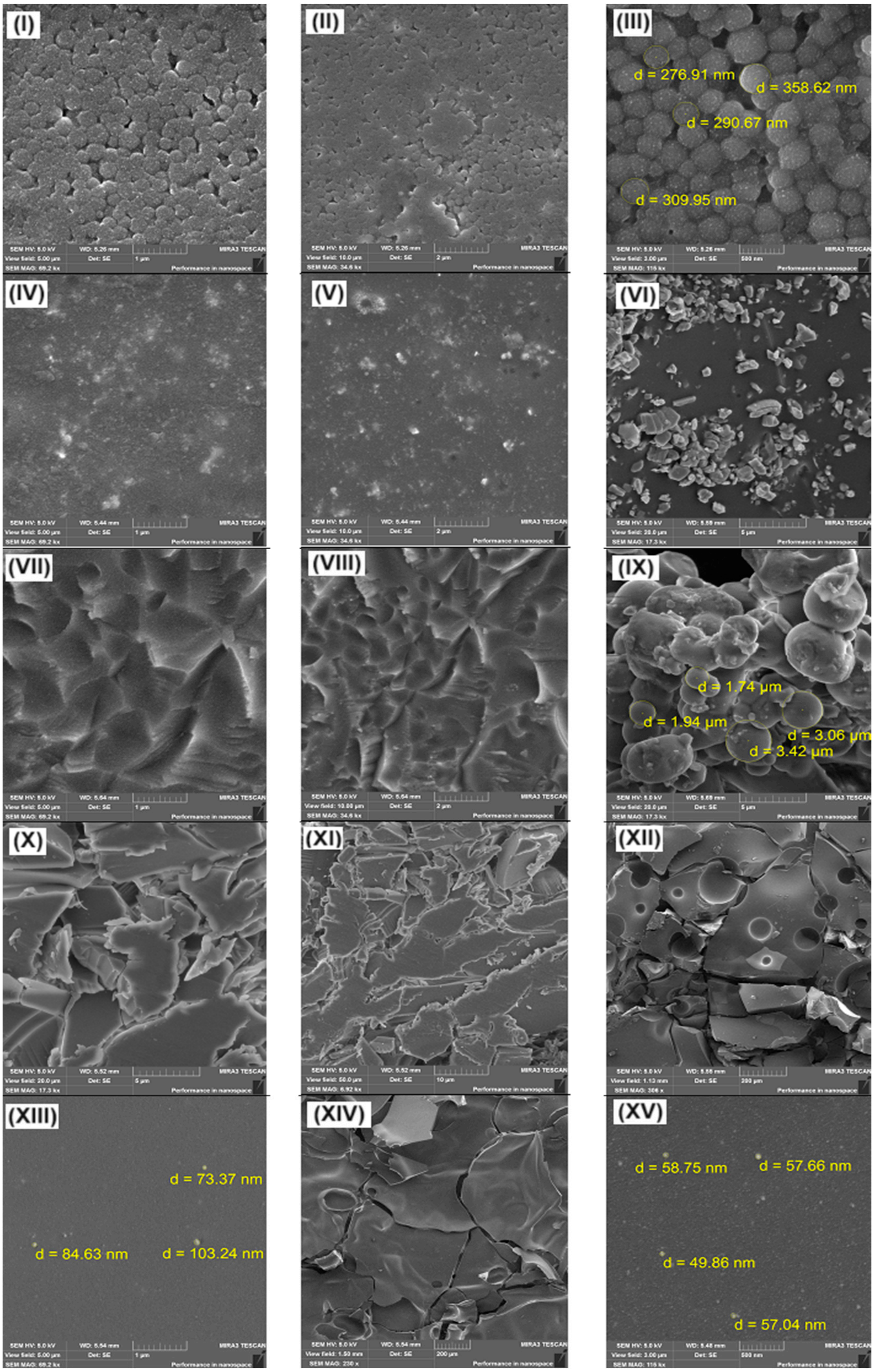

Figure S10. SEM image of a sample of the Cz-β-CD-Ag inclusion complex after heat treatment at 90°C (I, II, III), 160°C (IV, V, VI), 250°C (VII, VIII, IX), 315°C (X, XI, XII), 360°C (XIII, XIV, XV)

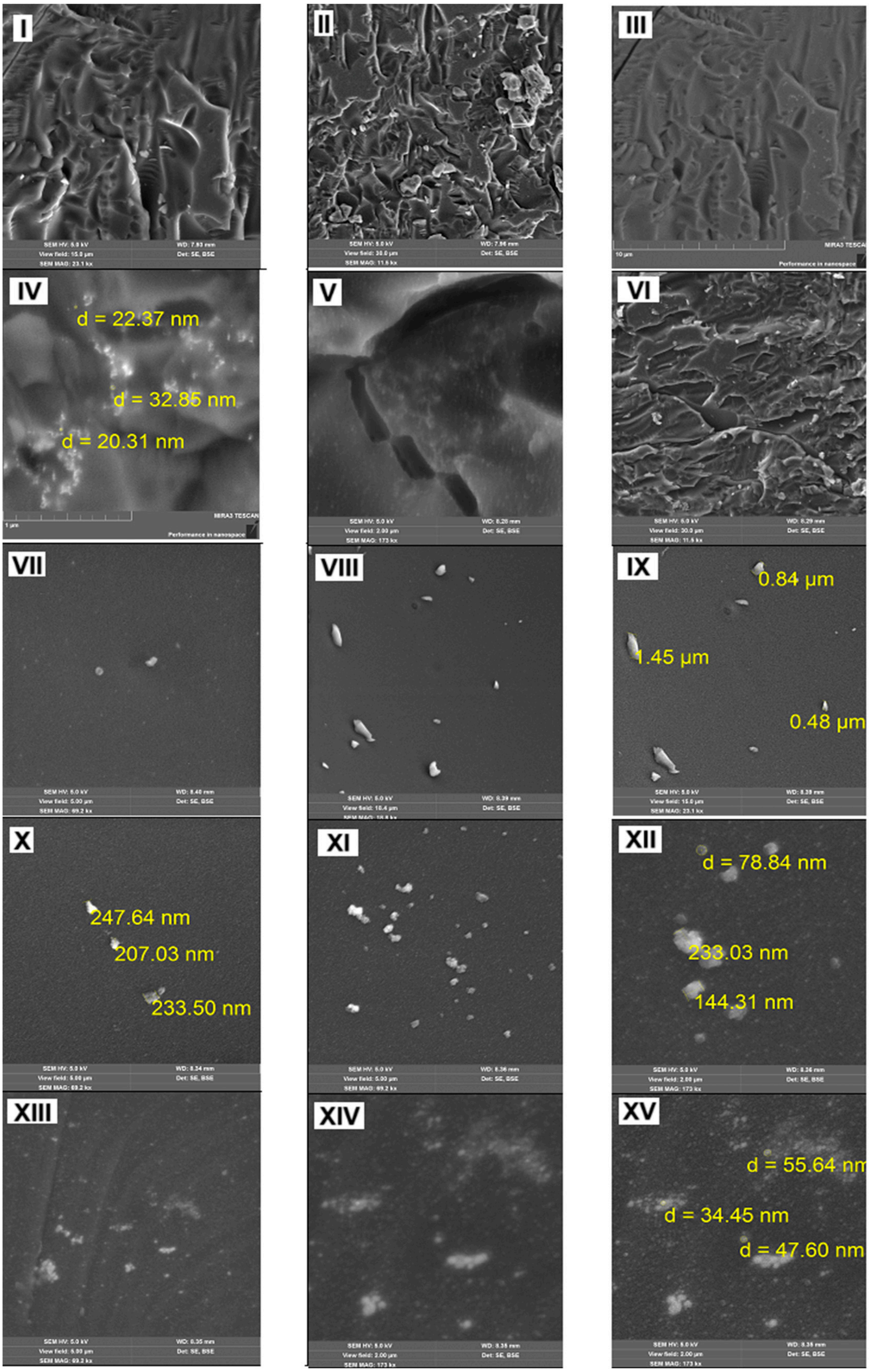

Figure S11. SEM image of a sample of the CzP-β-CD-Ag inclusion complex after heat treatment at 90°C I, II, III), 160°C (IV, V, VI), 250°C (VII, VIII, IX), 315°C (X, XI, XII), 360°C (XIII, XIV, XV)
